# Improved Confidence in a Confirmatory Stage by Application of Item-Based Pharmacometrics Model: Illustration with a Phase III Active Comparator-Controlled Trial in COPD Patients

**DOI:** 10.1007/s11095-022-03194-1

**Published:** 2022-03-01

**Authors:** Carolina Llanos-Paez, Claire Ambery, Shuying Yang, Misba Beerahee, Elodie L. Plan, Mats O. Karlsson

**Affiliations:** 1 Department of Pharmacy, Uppsala University, BMC, Box 580, 751 23 Uppsala, Sweden; 2 Clinical Pharmacology Modelling and Simulation, GlaxoSmithKline plc, London, UK

**Keywords:** chronic obstructive pulmonary disease, item response theory, mixed model repeated measures, non-linear mixed effect model, patient-reported outcomes

## Abstract

**Purpose:**

The current study aimed to illustrate how a non-linear mixed effect (NLME) model-based analysis may improve confidence in a Phase III trial through more precise estimates of the drug effect.

**Methods:**

The FULFIL clinical trial was a Phase III study that compared 24 weeks of once daily inhaled triple therapy with twice daily inhaled dual therapy in patients with chronic obstructive pulmonary disease (COPD). Patient reported outcome data, obtained by using The Evaluating Respiratory Symptoms in COPD (E-RS:COPD) questionnaire, from the FULFIL study were analyzed using an NLME item-based response theory model (IRT). The change from baseline (CFB) in E-RS:COPD total score over 4-week intervals for each treatment arm was obtained using the IRT and compared with published results obtained with a mixed model repeated measures (MMRM) analysis.

**Results:**

The IRT included a graded response model characterizing item parameters and a Weibull function combined with an offset function to describe the COPD symptoms-time course in patients receiving either triple therapy (n = 907) or dual therapy (n = 894). The IRT improved precision of the estimated drug effect compared to MMRM, resulting in a sample size of at least 3.64 times larger for the MMRM analysis to achieve the IRT precision in the CFB estimate.

**Conclusion:**

This study shows the advantage of IRT over MMRM with a direct comparison of the same primary endpoint for the two analyses using the same observed clinical trial data, resulting in an increased confidence in Phase III.

**Supplementary Information:**

The online version contains supplementary material available at 10.1007/s11095-022-03194-1.

## Introduction

The primary reason for Phase III clinical drug development failure is insufficient drug efficacy (55%), followed by safety (14%) and strategic reasons (14%) (1). Although the development of “ineffective” or “unsafe” compounds should stop during early stages of drug development, the proportion of failure owing to insufficient efficacy is currently larger in Phase III compared to Phase II (55% vs. 48%) (1). The causes of such failures may include not only insufficient drug efficacy but also insufficient knowledge of the treatment effect in the target population (2) leading to an insufficient sample size (e.g. sample size calculated from a commonly overestimated true treatment effect in Phase II (3)), and therefore high uncertainty in the efficacy estimate. A model-informed drug development (MIDD) decision-making framework has been proposed to increase Phase III trials probability of success through more precise estimates of the drug effect. This may result in an increased proportion of successful Phase III and IV trials (4).

Chronic Obstructive Pulmonary disease (COPD) is an inflammatory disease of the respiratory system, accounting for 54.9% of chronic respiratory diseases in 2017 (5). It was the third leading cause of death in 2016 and is projected to remain among the five leading causes of death by 2030 (6). The Global Initiative for Chronic Obstructive Lung Disease (GOLD) strategy (7) states that commonly used maintenance medications in COPD are short or long acting β2 agonists (SABA and LABA, respectively), short or long acting anticholinergic (SAMA and LAMA, respectively) or combination therapies with/without addition of inhaled corticosteroids (ICS). The GOLD strategy recommends that patients with advanced COPD and persistent symptoms who are at risk of exacerbations use an inhaled triple therapy (ICS/LABA/LAMA) (7). The Lung Function and Quality of life Assessment in COPD with Closed Triple Therapy (FULFIL) is the first Phase III study that compared a once-daily single-inhaler triple therapy with a twice-daily inhaled dual therapy (8). Patient-Reported Outcomes (PROs) from the FULFIL trial (8, 9) were obtained from different tools, such as the Evaluating Respiratory Symptoms in COPD (E-RS:COPD) (10), to compare the effect of fluticasone furoate/umeclidinium/vilanterol (FF/UMEC/VI) and budesonide/formoterol (BUD/FOR) on patient’s respiratory symptoms. PROs are an important aspect of drug development as they contribute directly with information on the patient’s own perceptions of their disease. The E-RS:COPD consists of 11 items from the 14-item Exacerbations of COPD Tool (EXACT), intended to capture information specifically related to respiratory symptoms. This tool has been derived through the application of item and Rasch model analysis (11).

The results of the statistical analysis of E-RS:COPD total score (RS-Total) from the FULFIL trial (8, 9) showed that FF/UMEC/VI significantly improved patient respiratory symptoms (assessed via E-RS:COPD tool) in comparison to BUD/FOR (9). This statistical analysis was performed using a mixed model repeated measures (MMRM) approach, a standard statistical method used in longitudinal clinical trials (12). This type of analysis may result in a loss of information and therefore a loss of precision in the efficacy estimate since MMRM analyzes the total score only, ignoring the contribution of each item in the questionnaire to the disease state. Moreover, in MMRM, time is handled as a discrete value rather than a continuous variable resulting in a further loss of information. To include all information from the data and thus increase power and precision to detect a drug effect, a MIDD approach can be applied to analyze PRO data such as RS-Total using a longitudinal non-linear mixed effect (NLME) analysis based on item-level data (i.e. item response theory based model - IRT) (13). In contrast to MMRM, IRT utilizes all components of the composite observations by relating its items to an underlying disease state that varies among individuals and changes with time. IRT thus utilizes all information captured by the questionnaire instead of only the total score as MMRM does.

In the planning of Phase III, different metrics are of interest for the design and analysis of trials such as the probability of study success or the probability of making a correct decision (14). An MIDD framework can provide a more informative approach to explore these metrics and therefore to improve late-stage clinical development productivity (4). This analysis aims to illustrate how a new methodology to analyze PRO data using an item-based pharmacometrics model would improve confidence in a confirmatory stage by comparing the precision around the primary efficacy endpoint obtained with IRT and MMRM.

## Materials and Methods

### Data and Patients

The FULFIL clinical trial (NCT02345161) was a Phase III, randomized, double-blind, double dummy, parallel-group, multicenter study that compared 24 weeks of once daily FF/UMEC/VI (100 μg/62.5 μg/25 μg) inhalation powder with twice daily BUD/FOR (400 μg/12 μg) in patients with COPD. Patients were randomized in a 1:1 ratio to FF/UMEC/VI or BUD/FOR. The study inclusion and exclusion criteria were reported elsewhere (8). In this analysis, nine patients were excluded from the intention to treat population for the following reasons: absence of E-RS:COPD score data for the whole study period (4 patients), dispensing errors (4 patients), and missing recorded time (1 patient). Daily data records were obtained by the completion of the E-RS:COPD tool using an electronic diary, which did not allow patients to skip individual items, although missing days (where patients did not provide answer for any of the items) were possible (10). The E-RS:COPD tool contains 11 items (a subset of the EXACT) that capture information related to respiratory symptoms of COPD (breathlessness, cough, sputum production, chest congestion and chest tightness), with seven items including five ordered categorical options and four items with four categories (Table I).

**Table I:** Content of the E-RS:COPD Tool

Item number	Item-level Construct	Score	Symptom Construct
7	Breathless today	0–4	Breathlessness
8	Breathless with activity	0–3	
9	Short of breath – personal care	0–4	
10	Short of breath – indoor activities	0–3	
11	Short of breath – outdoor activities	0–3	
2	Cough frequency	0–4	Cough and Sputum
3	Mucus quantity	0–3	
4	Difficulty with mucus	0–4	
1	Congestion	0–4	Chest Symptoms
5	Discomfort	0–4	
6	Tightness	0–4	

RS-Total is based on summation to yield ordinal-level scales with a range of 0–40

### IRT Development

The IRT was developed in two steps: 1) characterization of the item characteristic functions (ICFs) and 2) the development of the longitudinal model (a flow chart of the analysis is shown Supplementary Fig. 1).

#### Item Characteristics Functions

The properties of each item were determined by non-linear functions (the ICFs) linking the unobserved patient’s disease status, the latent variable θ, to the probability of giving a particular response for an item. Item parameters such as discrimination and difficulty parameters were estimated using an “independent occasion” approach (15). Under this approach, observations from each measurement occasion were treated as belonging to separate individuals, assuming a normal distribution with fixed mean and variance *N*(0,1) of θ at baseline, and estimated mean and variance *N*(μ,ω^2^) at later observations.

A logistic transformation was used to model each item with the probability of rating a score of at least *k* [P(y_ij_ ≥ *k*)] and the probability of rating exactly score *k* [P(y_ij_ = *k*)] as shown in Eq.1 and Eq. 2, respectively. These probabilities are functions of θ_*i*_, the latent variable of patient *i*, and the estimated fixed effect item parameters such as discrimination (a_*j*_) and difficulty (b_*j,k*_) parameters for item *j*; more specifically b_*j,k*_ is the difficulty parameter for the item-score *k*, and y_*ij*_ corresponds to the observed data (E-RS:COPD scores) for an individual *i* and an item *j*.

1 $$P\left({y}_{ij}\geq k\right)=\frac{e^{\left({a}_j\left({\uptheta}_i-{b}_{j,k}\right)\right)}}{1+{e}^{\left({a}_j\left({\uptheta}_i-{b}_{j,k}\right)\right)}}$$

2 $$P\left({y}_{ij}=k\right)=P\left({y}_{ij}\geq k\right)-P\left({y}_{ij}\geq k+1\right)$$

#### Longitudinal Model

In this step, ICF parameters were fixed to the values obtained in step 1, and a data reconciliation was needed to include each individual’s time-course data (original IDs). This was done since in step 1 each time point was treated as belonging to separate individuals. For the longitudinal model, the estimates of θ_*i*_ from the IRT, using ICF fixed parameters from step 1 were considered as the dependent variable (15) taking into account their uncertainty. These estimates of θ_*i*_ were obtained from an intermediate step where a large uncertainty for θ was considered to ensure that the total scores translated from the estimates of θ_*i*_ described the raw data well (as shown in Supplementary Fig. 2). The NONMEM control stream for this intermediate step to obtain θ_*i*_ for step 2 is available in the Supplementary material. A pre-specified Weibull function (Eq. 3) was used to describe changes in individual symptoms-time course (θ_*i*_) for both treatment arms. Parameters such as disease progression time (T_prog*i*_), maximum response (R_max*i*_) and baseline latent variable (θ_*i,t = 0*_) are subject-specific parameters with inter-individual variability (IIV), including a random variable with a mean of 0 and variance of ω^2^*.* T_progi_ was assumed to be log-normally distributed with IIV modeled using an exponential function, whereas all other parameters were assumed to be normally distributed with an additive IIV model. Time t is in years and γ is the gamma value that governs the steepness of the curve. Different parameters per treatment arm were considered. Additionally, effects of smoking status and geographical regions on θ_i,t = 0_ were included in the model as these covariates were considered in the MMRM analysis (9).

3 $${\uptheta}_i={\uptheta}_{i,t=0}+{R}_{maxi}\cdot \left(1-e\left(-{\left(\frac{\ln (2)}{T_{progi}}\cdot t\right)}^{\gamma}\right)\right)$$

### Internal Model Evaluation

Non-parametric ICF smooth plots were developed to assess ICF fit (16), and the predictive performance of the model was assessed by using Visual Predictive Check plots (VPCs). The 2.5th, 50th and 97.5th percentile of the observed data were compared to the 95%CI for the 2.5th, 50th and 97.5th percentiles of the simulated (N = 500) data. At this stage, further changes to the Weibull model were considered if it was deemed necessary.

### Clinical Endpoint

#### Clinical Endpoint Definition

The clinical endpoint was the change from baseline (CFB) in RS-Total over 4-week intervals for each treatment arm (FF/UMEC/VI and BUD/FOR). To provide context for the mean differences between groups and being able to compare changes in PRO scores between the treatment arms, a clinically meaningful CFB was defined as a change equal to or greater than a minimal clinically important difference (MCID) of 2 units (9). This means that a decrease of at least 2 points from baseline in RS-Total was deemed clinically significant.

#### Precision in Clinical Endpoint Estimate

**Fig. 1:**
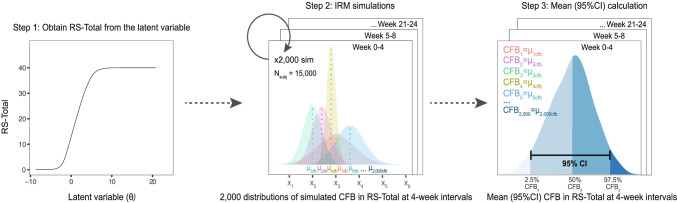
Schematic representation of the workflow for the simulations including parameter uncertainty. Distribution of CFB in RS-Total for each simulation considering 15,000 subjects and the mean (95%CI) CBF in RS-Total obtained from the average CFB of each simulation

The point estimate and the precision in the estimated clinical endpoint was obtained through model simulations, with inclusion of the uncertainty in the estimated longitudinal IRT parameters. RS-Total, linked to the individual patient disease status (θi), were simulated using the final IRT parameter estimates. The derived relationship between θ and RS-Total (Fig. 1) was used as a basis in the simulations. These stochastic simulations included parameter uncertainty from the estimated asymptotic variance-covariance matrix of the estimates by using the $PRIOR functionality in NONMEM. Specifically, the NWPRI subroutine was used where prior of fixed and random effects were assumed to be normally and inverse Wishart distributed, respectively. Degrees of freedom for the inverse Wishart distribution were calculated based on standard error (SE) of the obtained parameter estimates (17). RS-Total were simulated (N = 2000) over a period of 24 weeks for each treatment arm, using a large number of virtual subjects (Nsubj = 15,000 per arm). The individual average RS-Total at 4-week intervals was calculated and subtracted from the individual RS-Total at baseline. The distribution of CFB in RS-Total for each simulation (including 15,000 subjects) are illustrated in Fig. 1. The median, 2.5th, and 97.5th percentiles of the average CFB from each distribution were used to represent mean (95%CI) CFB in RS-Total (Fig. 1). These IRT derived values were compared with those (published values) obtained using the MMRM analysis (9).

#### Sample Size

The relative sample size (N) of a study analyzed using MMRM versus IRT can be calculated based on the precision (CI) of estimates for MMRM (95%CI length - CIMMRM) and IRT (95%CI length - CIIRT) for the same sample size as shown in Eq. 4. An N larger than one indicates that a MMRM analysis requires a larger study size to achieve the same precision as an IRT analysis.

4 $$N={\left(\frac{CI_{MMRM}}{CI_{IRT}}\right)}^2$$

### Software and Estimation Method

The software NONMEM (ICON Development Solutions, Ellicott City, Maryland) version 7.4.4 (18) was used for modeling (using the first-order conditional estimation method (step 2) plus Laplacian (step 1)) and simulation together with an Intel FORTRAN compiler and Perl-speaks-NONMEM (PsN, http://psn.sourceforge.net) version 5.1.0 (19). R software (The R Foundation for Statistical Computing) version 3.5.2 (20) and R packages, such as Xpose4 (http: //xpose.sourceforge.net, version 4.6.1) (21, 22) and Piraid (version 0.4) (23) were used for data management, graphical analysis, to produce summary statistics, and to examine the table outputs from NONMEM.

## Results

### Data and Patients

Data from 1801 patients (mean [standard deviation] age of 63.9 years [8.65], 43.8% smokers at study initiation) who received 24 weeks of either FF/UMEC/VI (n = 907) or BUD/FOR (n = 894) were included in this analysis. Baseline characteristics are shown in Table II. One-thousand six-hundred forty-three (91%) patients provided data up to at least week 24 with a median (range) missing days of 5 (0–101), whereas 158 patients (9%) stopped filling out the questionnaire after 84 (1–160) days with 3 (0–86) missing days.

**Table II:** Patient Characteristics at Baseline

Baseline characteristics	FF/UMEC/VI (n = 907)	BUD/FOR (n = 894)
Age (years)	64.2 (8.56)	63.6 (8.73)
Time with COPD (years)	< 1 y: 28 (3%)	< 1 y: 39 (4%)
	1–5 y: 325 (36%)	1–5 y: 334 (37%)
	5–10 y: 300 (33%)	5–10 y: 281 (31%)
	10–15 y: 165 (18%)	10–15 y: 147 (16%)
	≥ 15 y: 90 (10%)	≥ 15 y: 93 (10%)
FVC (L)	2.84 (0.80)	2.87 (0.79)
FEV_1_ (L)	1.25 (0.46)	1.24 (0.45)
Male (n)	675 (74%)	658 (74%)
Smoker (n)	396 (44%)	392 (44%)
COPD GOLD disease status	Moderate: 298 (33%)	Mild: 1 (0.1%)
	Severe: 501 (55%)	Moderate: 290 (32%)
	Very severe: 107 (12%)	Severe: 477 (53%)
		Very severe: 124 (14%)
RS-Total^b^	12.2 (5.85)	12.9 (5.96)

In this analysis, nine patients were excluded from the intention to treat population of the FULFIL clinical trial (NCT02345161) for the following reasons: absence of E-RS:COPD score data for the whole study period (4 patients), dispensing errors (4 patients), and missing recorded time (1 patient); ^b^ RS-Total was calculated as the mean value during baseline period defined as from day −14 to day −1

### IRT and Simulations Including Parameter Uncertainty

ICF parameters were estimated with good precision (Supplementary Table I). Item characteristic curves that illustrate the relationship between disease status and probability of giving a certain score for all items are shown in Supplementary Fig. 3. Addition of an offset drug effect value and correlation between θi,t = 0, Rmax and offset improved the description of data. The offset drug effect (Eq.5) value, that is different between treatment arms, was assumed to be normally distributed with *IIV modeled using an additive function.*

5 $${\uptheta}_i=\left\{\begin{array}{l}{\uptheta}_{i,t=0}\\ {}{\uptheta}_{i,t=0}+{R}_{maxi}\cdot \left(1-e\left(-{\left(\frac{\ln (2)}{T_{progi}}\cdot t\right)}^{\gamma}\right)\right)+{offset}_i\end{array}\right.\ {\displaystyle \begin{array}{l}\ t=0\\ {}\ t>0\end{array}}$$

**Fig. 2:**
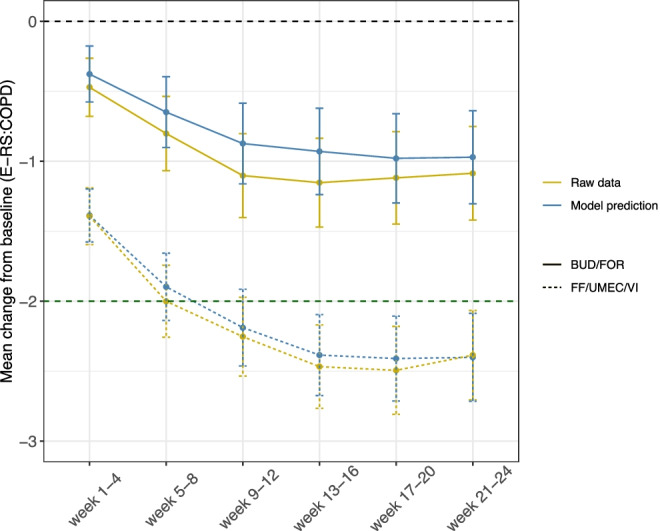
Mean (95%CI) CFB in RS-Total at 4-week intervals for observed data (yellow) and predicted values from the IRT (blue). Green dashed line indicates the MCID target

Different parameters per arm were estimated with a median (range) relative standard error (RSE) of 0.13 (0.02–0.75) (Table III). This model also showed an acceptable fit to the total score data for both treatment arms. Specifically, the model predicts satisfactorily the median observed total score data, as seen with agreement between the observed and simulated data in the VPC (Supplementary Fig. 4). Goodness of fit plots are shown in Supplementary Fig. 5, and longitudinal model parameter estimates are shown in Table III. While typical T_prog_ was similar between the two arms, more negative R_max_ (−0.31 vs. -0.16) and offset (−0.27 vs. -0.08) values in the FF/UMEC/VI arm, indicated higher benefit of the FF/UMEC/VI treatment to the patient compared to the BUD/FOR arm. Model predicted and raw data for CFB in RS-Total at 4-week intervals are shown in Fig. 2. According to the IRT, in the BUD/FOR arm, the CFB in RS-Total score did not achieve the MCID at any time point, whereas in the FF/UMEC/VI arm, the mean CFB in RS-Total achieved the MCID from week 9 onwards, which is in agreement with the observed data (Fig. 2). NONMEM control stream and snippet of data for both models ICFs and longitudinal are provided in the Supplementary material.

**Table III:** Parameter Estimates for the Longitudinal Model

Parameter estimates	FF/UMEC/VI	BUD/FOR
	Value (RSE)	Value (RSE)
θ_t = 0_ (unitless)	0.33 (0.23)	0.29 (0.28)
T_prog_ (year)	0.08 (0.03)	0.08 (0.03)
R_max_ (unitless)	−0.31 (0.11)	−0.16 (0.25)
γ (unitless)	9.27 (0.13)	16.9 (0.75)
Offset (unitless)	−0.27 (0.08)	−0.08 (0.30)
ω^2^ θ_t = 0_	1.09 (0.03)	1.47 (0.03)
ω^2^ T_prog_	0.45 (0.03)	0.46 (0.03)
ω^2^ R_max_	0.89 (0.04)	0.98 (0.06)
ω^2^ Offset	0.37 (0.05)	0.42 (0.05)
ω^2^ R_max_ ~ ω^2^ Offset	11% (0.24)	–
ω^2^ θ_t = 0_ ~ ω^2^ Offset	−12% (0.20)	−10% (0.27)
ω^2^ R_max_ ~ ω^2^ θ_t = 0_	–	−13% (0.17)
Residual unexplained variability	0.32 (0.02)	
Smoking effect on θ_t = 0_ (additive)	0.13 (0.62)	
Region 1 on θ_t = 0_ (additive)	−0.64 (0.13)	
Region 3 on θ_t = 0_ (additive)	−0.61 (0.13)	
Region 4 on θ_t = 0_ (additive)	−0.20 (0.40)	
Region 5 on θ_t = 0_ (additive)	−0.41 (0.27)	
Region 6 on θ_t = 0_ additive)	−0.98 (0.12)	

Region 1 (21%): Germany, Greece, Italy; Region 2 (24%): Russian Federation, Ukraine; Region 3 (21%): Bulgaria, Hungary, Romania, Slovakia; Region 4 (18%): Czech Republic, Estonia, Poland; Region 5 (6%): China, Republic of Korea; Region 6 (10%): Mexico; θ_t = 0_: baseline latent variable; T_prog_: disease progression time; R_max_: maximum response; RSE: relative standard error (for omega and sigma RSEs are reported on the approximate standard deviation scale); ω^2^: variance describing inter-individual variability

**Fig. 3:**
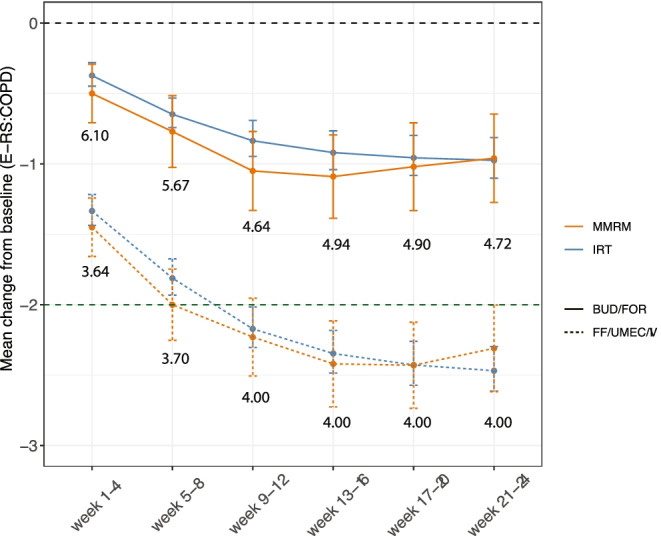
Mean (95%CI) for CFB in RS-Total at 4-week intervals simulated with IRT (blue) and published MMRM values (red). Green dashed line indicates the MCID target. Values correspond to the relative sample size (Eq. 4)

Based on simulations including parameter uncertainty, the IRT considerably improved the precision of the drug effect compared to the MMRM at every time point (Fig. 3 and Table IV). At the end of treatment (week 21–24), the mean (95%) CFB in RS-Total was −2.47 (−2.61, −2.30) with IRT compared to −2.31 (−2.62, −2.00) with MMRM in the FF/UMEC/VI arm, and the mean (95%) CFB in RS-Total was −0.97 (−1.10, −0.81) with IRT compared to −0.96 (−1.27, −0.65) with MMRM in the BUD/FOR arm. Furthermore, a relative sample size (N; obtained using Eq. 4) of 4.00 (FF/UMEC/VI) and 4.72 (BUD/FOR) times larger would be required in the MMRM analysis to achieve the precision obtained with the IRT analysis at the end of the study (week 21–24). Sample size requirements at each week interval are shown in Fig. 3.

**Table IV:** Mean (95%CI) Change from Baseline (CFB) in RS-Total for IRM and MMRM at 4-week Intervals

	FF/UMEC/VI		BUD/FOR	
Week interval	CFB in RS-Total. Mean (95% CI) [SE]		CFB in RS-Total. Mean (95% CI) [SE]	
	MMRM	IRM	MMRM	IRM
1–4	−1.45 (−1.66, −1.24)	−1.33 (−1.44, −1.22)	−0.50 (−0.71, −0.29)	−0.37 (−0.45, −0.28)
	[0.11]	[0.06]	[0.11]	[0.04]
5–8	−2.00 (−2.25, −1.75)	−1.81 (−1.93, −1.67)	−0.77 (−1.02, −0.52)	−0.65 (−0.74, −0.53)
	[0.13]	[0.07]	[0.13]	[0.05]
9–12	−2.23 (−2.51, −1.95)	−2.17 (−2.30, −2.02)	−1.05 (−1.33, −0.77)	−0.84 (−0.95, −0.69)
	[0.14]	[0.07]	[0.14]	[0.07]
13–16	−2.42 (−2.73, −2.11)	−2.35 (−2.49, −2.18)	−1.09 (−1.39, −0.79)	−0.92 (−1.04, −0.77)
	[0.15]	[0.08]	[0.15]	[0.07]
17–20	−2.43 (−2.74, −2.12)	−2.43 (−2.57, −2.26)	−1.02 (−1.33, −0.71)	−0.96 (−1.08, −0.80)
	[0.16]	[0.08]	[0.16]	[0.07]
21–24	−2.31 (−2.62, −2.00)	−2.47 (−2.61, −2.30)	−0.96 (−1.27, −0.64)	−0.97 (−1.10, −0.81)
	[0.16]	[0.08]	[0.16]	[0.07]

## Discussion

Using data from a Phase III study, a longitudinal IRT improved the precision in the efficacy endpoint compared to MMRM. The use of NLME analysis based on item-level data (IRT) was recently proposed as an alternative to MMRM for evaluation of efficacy in the analysis of data from a Phase II study, where IRT improved the precision of the estimated drug effect considerably in comparison to MMRM (24). The benefits of using an IRT over other statistical methods such as least-square mean analysis have already been demonstrated, highlighting its higher power to detect a drug effect (13, 25). This could be explained by the fact that IRT is a more informative approach that not only describe the longitudinal aspects of the data but also accounts for information from the scores given to the individual items of the questionnaire. In contrast, MMRM ignores this information in the data since i) it is an analysis of total score data only and ii) each visit is modeled independently as a factor with earlier time points contributing less information about the end-of-treatment response than what is the case for NLME model. MMRM analysis is designed for robustness rather than efficiency (12), hence it could be expected that IRT analysis is more efficient than MMRM. However, this study provides an insight into the magnitude of improvement in precision that an IRT model may provide and the consequences this can have on the required sample size.

The primary objective of the Phase III study analysis is the confirmation of efficacy (and safety) profiles of an investigational new drug. Overall, an increased precision around the efficacy estimate was observed with IRT compared to MMRM. This analysis highlights that a smaller sample size would have been required to confirm the drug effect if a NLME model-based approach were to have been used instead of the MMRM approach. A median relative sample size over the 4-week intervals of 4.0 (FF/UMEC/VI) and 4.9-fold (BUD/FOR) larger would have been required for the MMRM analysis to achieve the IRT precision. These values are comparable with *a 3.5-fold smaller study size with IRT compared to MMRM analysis using RS-Total data from a Phase II trial* (24). This is particularly important in cases where a large number of patients cannot be recruited for pivotal trials (e.g. rare diseases) with presumably a clearer benefit of using the IRT approach due to the increased utilization of the information contained in the item-level data. Thus, the increased precision in the efficacy estimate with IRT can lead to a higher probability of making a correct decision which appears important in light of the many failures in Phase III attributed to underpowered trials.

This analysis illustrates how simulations using an IRT with parameter uncertainty included can be used to obtain the precision of the primary efficacy endpoint. A large number of virtual subjects (15,000) were used in the simulations to make sure that uncertainty comes mainly from the model parameter estimates rather than the sample size. The SEs associated with each parameter estimates were obtained from the variance-covariance matrix in NONMEM. To further investigate how SEs obtained from different techniques such as bootstrap or sampling importance resampling would have impacted the precision in the efficacy endpoint was not in the scope of this study; however, it would be of interest to investigate how this would affect the calculated sample size.

This analysis is not exempt of limitations, which are described as follow. A predetermined (decided before start analyzing the data) longitudinal model (Weibull function) was considered; however, the addition of an extra parameter (offset) was required to describe the rapid onset of bronchodilator effects in both treatment arms, and thus improve the predictive performance of the model (based on the VPC plot). The absence of this parameter in the model would have led to a poorer description of the data, which is not necessarily related to a worse or better precision and/or relative sample size (26). The authors acknowledge that, in order to use a pharmacometric model as primary analysis in confirmatory trials, this model need to be pre-specified to avoid the risk of type 1 error inflation due to multiple testing during model building. One benefit of MMRM in this aspect is its flexibility and the fact that can adequately be pre-specified, though some assumptions are still required (12). Furthermore, in this analysis, model uncertainty (e.g. uncertainty from the structural part of model or from the random effects) and its impact on the precision around the efficacy endpoint was also not investigated. To mitigate model uncertainty in a NLME model-based analysis, it has been proposed to conduct model averaging (27, 28). Model averaging would cover the model space by assigning a goodness-of-fit derived weight to the different proposed structural models, thereby including model uncertainty for a pre-specified analysis. Lastly, it can be argued that a simpler NLME model for the total score as one continuous endpoint could achieve similar results, in terms of precision and power, than an IRT-based model analysis when assessing treatment effect on the CFB of total scores. A formal comparison between these two types of analyses was not performed in this study; however, previous work has suggested that IRT-based models are more informative and require sample size that are approximately 20–40% smaller than analysis of total score data (13, 29–31). Despite these limitations, this analysis shows the advantage of NLME analysis with a direct comparison of the same primary endpoint for the two methods (IRT and MMRM) using observed clinical trial data rather than focusing on drug effect parameters which are often unobserved. Based on simulations, it has been shown already that a NLME analysis can be more powerful than MMRM in some (albeit not all) scenarios (32).

## Conclusions

The positive impact of using a NLME model-based approach in decision-making during drug development has already been shown (4, 33). This analysis shows the advantage of using a NLME model based on item level data over a standard approach used today in drug development (MMRM) for the same endpoint, increasing the precision in the efficacy estimate and thereby significantly reducing the required sample size to confirm drug effect.

ACKNOWLEDGMENTS AND DISCLOSURES.

The authors would like to acknowledge Maggie Tabberer for her contributions to the data analysis and interpretation. Trademarks are owned by or licensed to EVIDERA. CL-P, ELP and MOK declare that they have no conflict of interest. CA, SY and MB are GSK employees and hold GSK shares.

## Supplementary Information

ESM 1 (DOCX 5423 kb)
