# Supplementary material for: Improved Confidence in a Confirmatory Stage by Application of Item-Based Pharmacometrics Model: Illustration with a Phase III Active Comparator-Controlled Trial in COPD Patients
Source: Pharm Res. 2022 Mar 1;39(8):1779–87. doi: 10.1007/s11095-022-03194-1 (PMC9314306; doi:10.1007/s11095-022-03194-1)

Improved confidence in a confirmatory stage by application of item-based pharmacometrics model: illustration with a Phase III active comparator-controlled trial in COPD patients

Carolina Llanos-Paez^1^, Claire Ambery^2^, Shuying Yang^2^, Misba Beerahee^2^, Elodie L. Plan^1^, Mats O. Karlsson^1*^

^1^ Department of Pharmacy, Uppsala University, Uppsala, Sweden.

^2^ Clinical Pharmacology Modelling and Simulation, GlaxoSmithKline plc., London, UK.

***Corresponding author:** Mats O. Karlsson, Department of Pharmacy, Uppsala University, BMC, Box 580, 751 23 Uppsala, Sweden; +4618-471 4105; mats.karlsson@farmaci.uu.se

**Keywords:** chronic obstructive pulmonary disease; mixed model repeated measures; non-linear mixed effect model; item response theory, patient-reported outcomes

Supplementary information

**Table 1** Item characteristic parameter estimates (Step 1)

| **Parameter** | **Mean (RSE)** | **Parameter** | **Mean (RSE)** |
| --- | --- | --- | --- |
| DIS_i1 | 1.79 (0.02) | DIS_i8 | 1.47 (0.02) |
| DIF1_i1 | -1.03 (0.04) | DIF1_i8 | -1.91 (0.03) |
| DIF2_i1 | 2.03 (0.02) | DIF2_i8 | 1.95 (0.02) |
| DIF2_i1 | 1.96 (0.02) | DIF2_i8 | 2.05 (0.02) |
| DIF4_i1 | 1.98 (0.03) | DIF4_i8 | 0 fix |
| DIS_i2 | 1.14 (0.02) | DIS_i9 | 2.05 (0.02) |
| DIF1_i2 | -2.29 (0.03) | DIF1_i9 | -0.65 (0.05) |
| DIF2_i2 | 2.74 (0.02) | DIF2_i9 | 1.49 (0.02) |
| DIF3_i2 | 2.54 (0.02) | DIF3_i9 | 1.51 (0.02) |
| DIF4_i2 | 2.74 (0.02) | DIF4_i9 | 1.76 (0.03) |
| DIS_i3 | 1.10 (0.02) | DIS_i10 | 2.58 (0.02) |
| DIF1_i3 | -1.92 (0.03) | DIF1_i10 | -1.16 (0.03) |
| DIF2_i3 | 5.76 (0.02) | DIF2_i10 | 1.51 (0.02) |
| DIF3_i3 | 2.96 (0.03) | DIF3_i10 | 1.64 (0.02) |
| DIF4_i3 | 0 fix | DIF4_i10 | 0 fix |
| DIS_i4 | 1.47 (0.02) | DIS_i11 | 2.16 (0.02) |
| DIF1_i4 | -0.99 (0.04) | DIF1_i11 | -1.65 (0.03) |
| DIF2_i4 | 1.78 (0.02) | DIF2_i11 | 1.45 (0.02) |
| DIF3_i4 | 1.60 (0.02) | DIF3_i11 | 1.71 (0.02) |
| DIF4_i4 | 1.69 (0.02) | DIF4_i11 | 0 fix |
| DIS_i5 | 2.32 (0.02) | DIS_i12 | 1.97 (0.02) |
| DIF1_i5 | -0.74 (0.04) | DIF1_i12 | -1.30 (0.03) |
| DIF2_i5 | 1.67 (0.02) | DIF2_i12 | 1.99 (0.02) |
| DIF3_i5 | 1.85 (0.02) | DIF3_i12 | 1.84 (0.02) |
| DIF4_i5 | 1.83 (0.03) | DIF4_i12 | 1.71 (0.03) |
| DIS_i6 | 2.20 (0.02) | DIS_i13 | 1.31 (0.02) |
| DIF1_i6 | -0.71 (0.05) | DIF1_i13 | -0.34 (0.09) |
| DIF2_i6 | 1.65 (0.02) | DIF2_i13 | 1.87 (0.02) |
| DIF3_i6 | 1.86 (0.02) | DIF3_i13 | 2.01 (0.02) |
| DIF4_i6 | 1.93 (0.03) | DIF4_i13 | 1.91 (0.03) |
| DIS_i7 | 2.64 (0.02) | DIS_i14 | 1.47 (0.02) |
| DIF1_i7 | -1.60 (0.03)0 | DIF1_i14 | -0.50 (0.06) |
| DIF2_i7 | 1.70 (0.02) | DIF2_i14 | 1.72 (0.02) |
| DIF3_i7 | 1.83 (0.02) | DIF3_i14 | 1.91 (0.02) |
| DIF4_i7 | 1.59 (0.02) | DIF4_i14 | 0 fix |
| θ at time = 0 (baseline) | 0 fix | IIV on θ at time = 0 (baseline) | 1 fix |
| θ at time ≠ 0 | -0.34 (0.09) | IIV on θ at time ≠ 0 | 1.61 (0.04) |

DIS: discrimination parameter; DIF: difficulty parameter; IIV: interindividual variability; θ: latent variable.

**Fig. 1** Flow chart of the analysis describing the steps including in the IRT development and how the longitudinal model was performed. Simulations including parameter uncertainty were performed using the final model

**
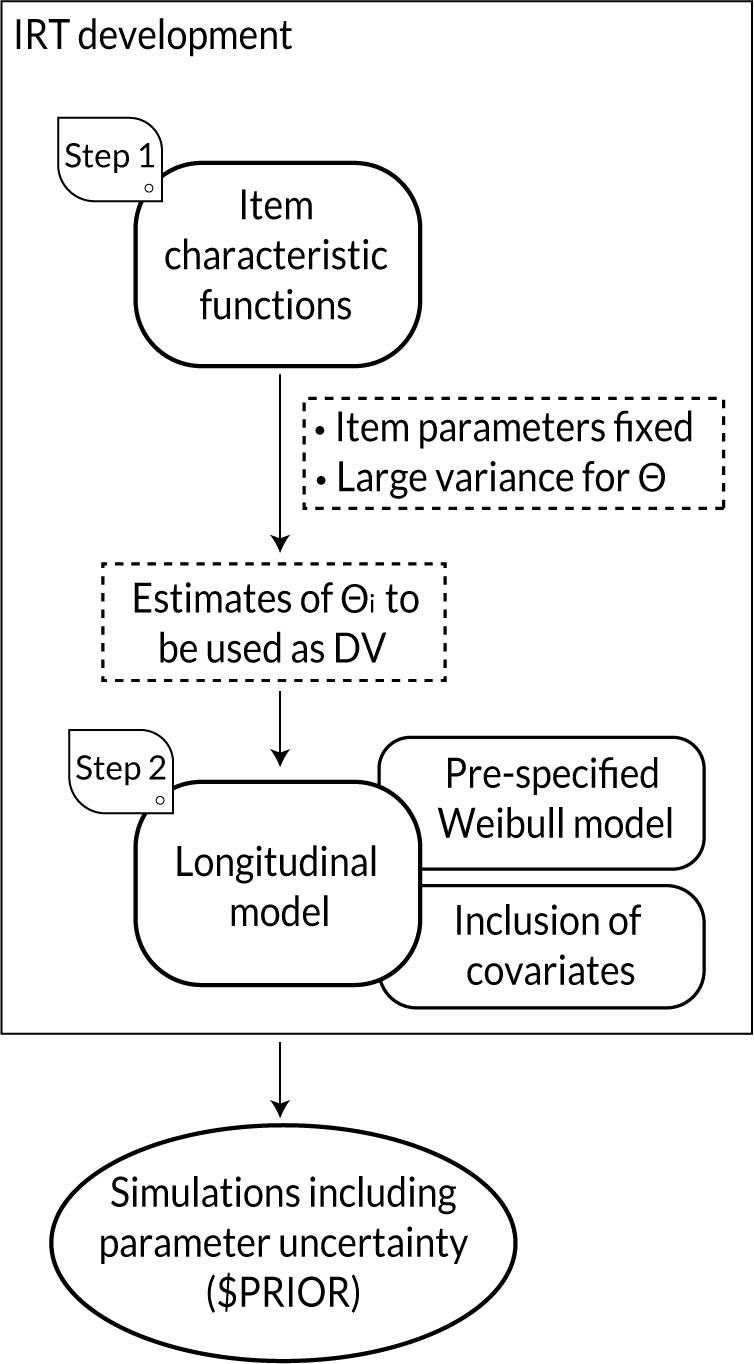
**

**Fig. 2** Comparison between total scores from the raw dataset and total scores derived from the estimates of θ_i_ in the intermediate step for both treatment arms. Estimates of θ_i_ were used as the dependent variable (DV) in step 2.

**
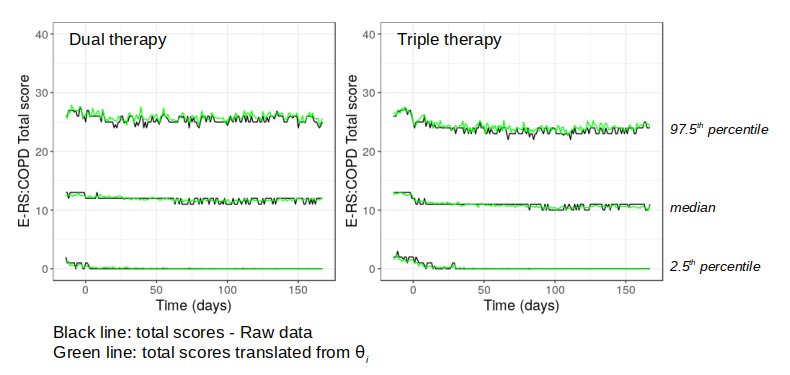
**

**Fig. 3** Item Characteristic curves for all 14 items showing the relationship between patient’s disease status (θ) and probability of giving a certain score.


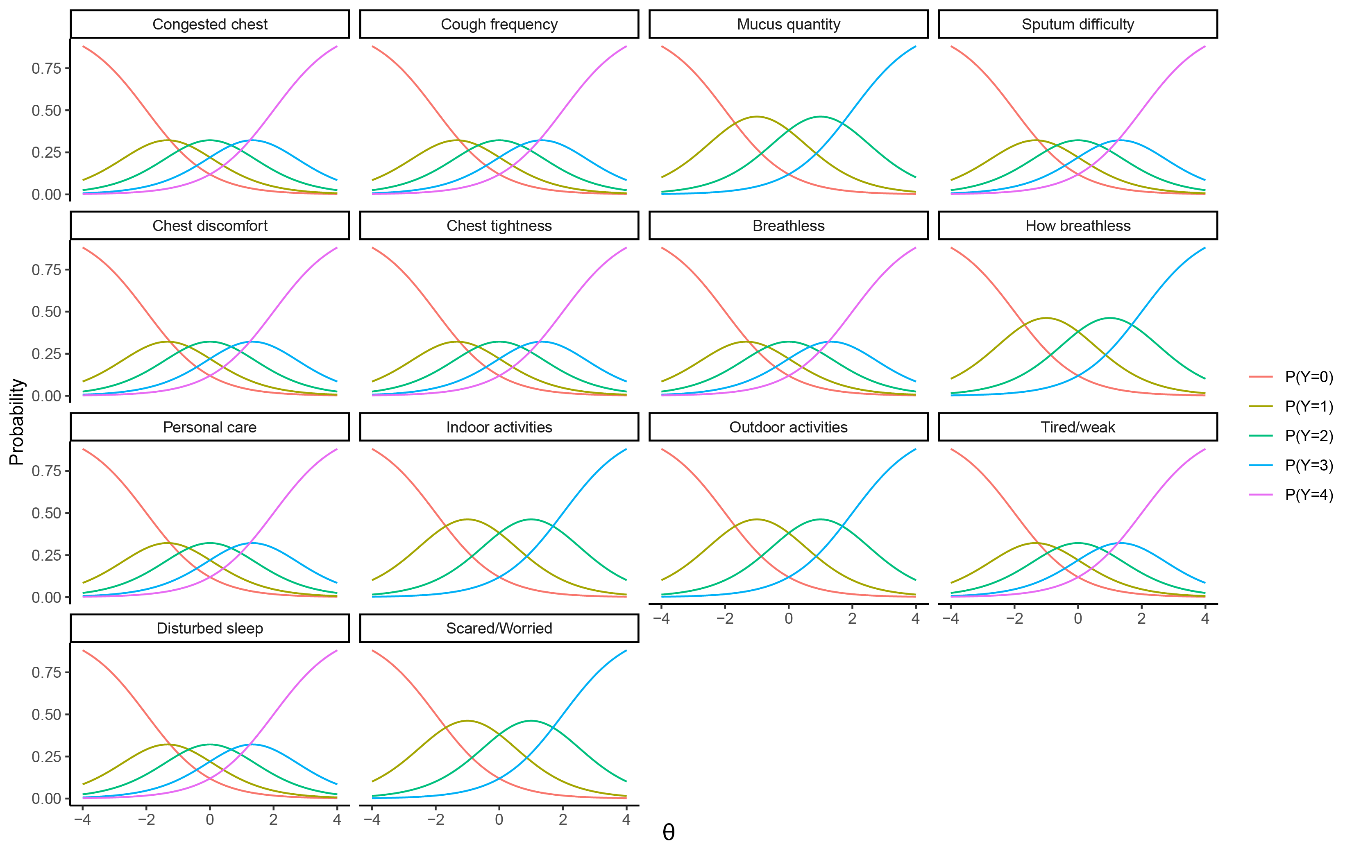


**Fig. 4** Visual predictive check for the E-RS:COPD total score in FF/UMEC/VI (a) and BUD/FOR (b) arms. Lines are the 2.5^th^, 50^th^ and 97.5^th^ percentile of the observed data, and grey areas are the corresponding 95% confidence interval from model simulations (N=500).


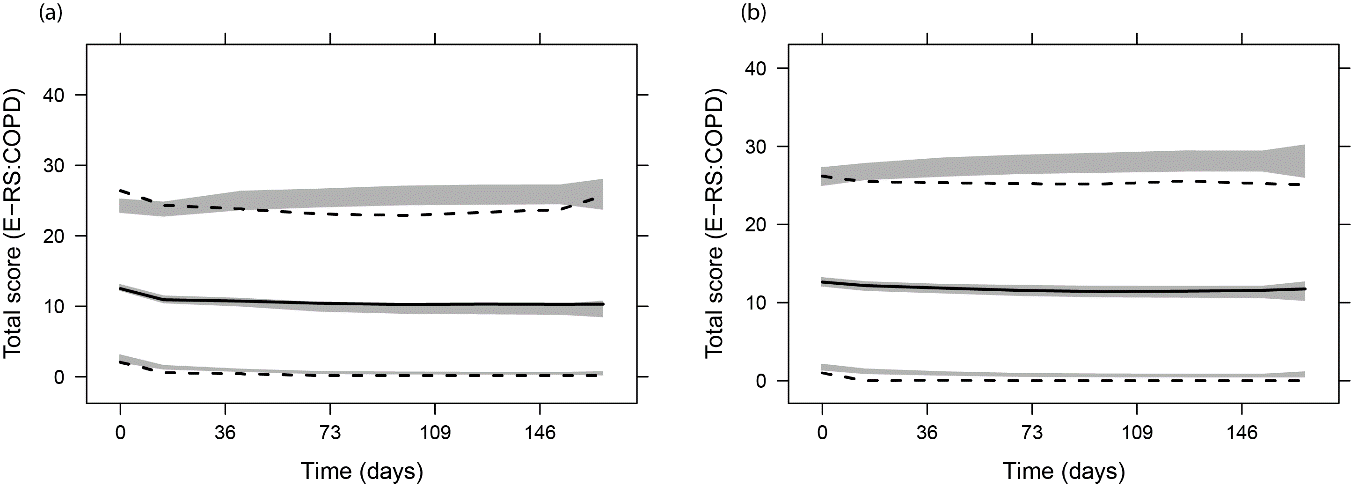


**Fig. 5** Goodness of fit plots (a) latent variable vs. individual predicted (IPRED); (b) latent variable vs. population predicted (PRED); (c) population conditional weighted residuals (CWRES) vs. individual predicted (IPRED); (d) CWRES vs. subject identification.


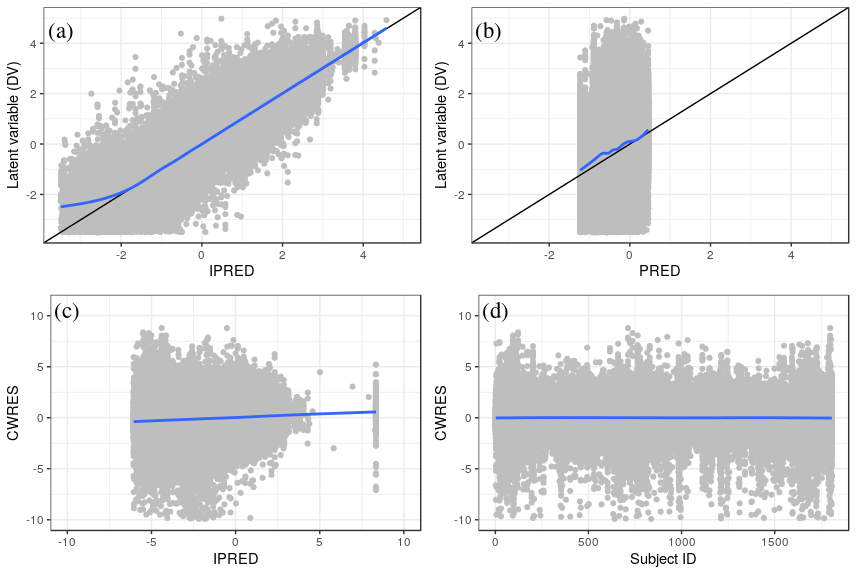


**NONMEM control stream and snippet of the data for the ICFs (step 1)**

$SIZES MAXFCN=100000000 NO=25000

$PROBLEM COPD PRO data modelling: ICC parameters

$INPUT OID TRT_GRP ITEM DV COUNTRY TOTAL ADDDAY EVID BDAY TIME ID

; time=newday-1, so that time starts at 0

; (new day is same as day, except for ADDDAY rows, see below)

; items 3, 8, 10, 11, 14 have DV 0-3; and the rest 0-4

; OID is the original ID number

; total is a flag indicating that item 99 is total score

; add day is a flag indicating that a day was added into the study (due to missing data); DV is -99 then

; BDAY is baseline time (BDAY=1 for positive time values, BDAY=2 for negative time values (screening period) BDAY=0 no baseline values).

; Most patients start at day 1, but some don’t so BDAY is a flag to indicate the first day of treatment for each patient (baseline)

; ID is a new ID at each time point (Independent occasion approach for ICC estimates)

$DATA study2_bytime_20200707.csv

IGNORE=@

IGNORE=(ADDDAY==1); ignore days that were added in

IGNORE=(TOTAL==1); ignore total score rows

IGNORE=(EVID==4); no MM elements included

IGNORE=(TIME<0); ignore screening period

$PRED

; --------- (re)set the PDV --------------------------

IF(NEWIND.NE.2.OR.BDAY.EQ.1) THEN; first row of a subject or an item

IF(EVID==0) TMPDV=DV ; first PDV=DV

NREC=1

OLDTIME=TIME

ENDIF

XNRC = NREC

IF(TIME>OLDTIME) NREC=NREC+1

IF(EVID==0) PDV=TMPDV

;------------ item parameter selection ----------------------

IF(ITEM==1) THEN

DIS = THETA(1)

DIF1 = THETA(2)

DIF2 = THETA(3)

DIF3 = THETA(4)

DIF4 = THETA(5)

ENDIF

IF(ITEM==2) THEN

DIS = THETA(6)

DIF1 = THETA(7)

DIF2 = THETA(8)

DIF3 = THETA(9)

DIF4 = THETA(10)

ENDIF

IF(ITEM==3) THEN

DIS = THETA(11)

DIF1 = THETA(12)

DIF2 = THETA(13)

DIF3 = THETA(14)

DIF4 = THETA(15) ; no need, only 0-3

ENDIF

IF(ITEM==4) THEN

DIS = THETA(16)

DIF1 = THETA(17)

DIF2 = THETA(18)

DIF3 = THETA(19)

DIF4 = THETA(20)

ENDIF

IF(ITEM==5) THEN

DIS = THETA(21)

DIF1 = THETA(22)

DIF2 = THETA(23)

DIF3 = THETA(24)

DIF4 = THETA(25)

ENDIF

IF(ITEM==6) THEN

DIS = THETA(26)

DIF1 = THETA(27)

DIF2 = THETA(28)

DIF3 = THETA(29)

DIF4 = THETA(30)

ENDIF

IF(ITEM==7) THEN

DIS = THETA(31)

DIF1 = THETA(32)

DIF2 = THETA(33)

DIF3 = THETA(34)

DIF4 = THETA(35)

ENDIF

IF(ITEM==8) THEN

DIS = THETA(36)

DIF1 = THETA(37)

DIF2 = THETA(38)

DIF3 = THETA(39)

DIF4 = THETA(40) ; no need, only 0-3

ENDIF

IF(ITEM==9) THEN

DIS = THETA(41)

DIF1 = THETA(42)

DIF2 = THETA(43)

DIF3 = THETA(44)

DIF4 = THETA(45)

ENDIF

IF(ITEM==10) THEN

DIS = THETA(46)

DIF1 = THETA(47)

DIF2 = THETA(48)

DIF3 = THETA(49)

DIF4 = THETA(50) ; no need, only 0-3

ENDIF

IF(ITEM==11) THEN

DIS = THETA(51)

DIF1 = THETA(52)

DIF2 = THETA(53)

DIF3 = THETA(54)

DIF4 = THETA(55) ; no need, only 0-3

ENDIF

IF(ITEM==12) THEN

DIS = THETA(56)

DIF1 = THETA(57)

DIF2 = THETA(58)

DIF3 = THETA(59)

DIF4 = THETA(60)

ENDIF

IF(ITEM==13) THEN

DIS = THETA(61)

DIF1 = THETA(62)

DIF2 = THETA(63)

DIF3 = THETA(64)

DIF4 = THETA(65)

ENDIF

IF(ITEM==14) THEN

DIS = THETA(66)

DIF1 = THETA(67)

DIF2 = THETA(68)

DIF3 = THETA(69)

DIF4 = THETA(70) ; no need, only 0-3

ENDIF

;----------------- hidden variable model -------------------

IF(TIME.EQ.0) THEN

BASELINE=THETA(71)+ETA(1)

ELSE

BASELINE=THETA(72)+ETA(2)

ENDIF

PSI=BASELINE ; COPD disease severity

;------ 2 parameter logit model implementation (4/5 states) ------

;------ constrain different states to be >= than the previous -----

DIFS1 = DIF1

DIFS2 = DIFS1+DIF2

DIFS3 = DIFS2+DIF3

DIFS4 = DIFS3+DIF4 IF(ITEM==3.OR.ITEM==8.OR.ITEM==10.OR.ITEM==11.OR.ITEM==14) DIFS4 = 0

; ------ probabilities for Y greater than 1 etc -------------

PGE1 = EXP(DIS*(PSI-DIFS1))/(1+EXP(DIS*(PSI-DIFS1)))

PGE2 = EXP(DIS*(PSI-DIFS2))/(1+EXP(DIS*(PSI-DIFS2)))

PGE3 = EXP(DIS*(PSI-DIFS3))/(1+EXP(DIS*(PSI-DIFS3)))

PGE4 = EXP(DIS*(PSI-DIFS4))/(1+EXP(DIS*(PSI-DIFS4)))

IF(ITEM==3.OR.ITEM==8.OR.ITEM==10.OR.ITEM==11.OR.ITEM==14) PGE4 = 0

; ------ probabilities for Y=0 etc --------------------------

P0 = 1-PGE1

P1 = PGE1-PGE2

P2 = PGE2-PGE3

P3 = PGE3-PGE4

IF(ITEM==3.OR.ITEM==8.OR.ITEM==10.OR.ITEM==11.OR.ITEM==14) P3 = PGE3

P4 = PGE4

IF(ITEM==3.OR.ITEM==8.OR.ITEM==10.OR.ITEM==11.OR.ITEM==14) P4 = 0

IF(DV==0) Y=P0

IF(DV==1) Y=P1

IF(DV==2) Y=P2

IF(DV==3) Y=P3

IF(DV==4) Y=P4

IPRED = (P0*0)+(P1*1)+(P2*2)+(P3*3)+(P4*4)

RES = DV - IPRED

;----- get XDV ------------------------------------

XDV=0

IF (EVID==0) XDV = PDV*10 + DV

;----- remember TIME, DV --------------------------

OLDTIME=TIME

IF(EVID==0.AND.ITEM.NE.99) TMPDV = DV

$THETA (0,1.79211) FIX ; 1. DIS_i1

$THETA -1.03362 FIX ; 2. DIF1_i1

$THETA (0,2.03489,1000000) FIX ; 3. DIF2_i1

$THETA (0,1.95689,1000000) FIX ; 4. DIF3_i1

$THETA (0,1.98008,1000000) FIX ; 5. DIF4_i1

$THETA (0,1.13922) FIX ; 6. DIS_i2

$THETA -2.29355 FIX ; 7. DIF1_i2

$THETA (0,2.74145,1000000) FIX ; 8. DIF2_i2

$THETA (0,2.53703,1000000) FIX ; 9. DIF3_i2

$THETA (0,2.74127,1000000) FIX ; 10. DIF4_i2

$THETA (0,1.10396) FIX ; 11. DIS_i3

$THETA -1.91627 FIX ; 12. DIF1_i3

$THETA (0,5.75838,1000000) FIX ; 13. DIF2_i3

$THETA (0,2.96354,1000000) FIX ; 14. DIF3_i3

$THETA 0 FIX ; 15. DIF4_i3

$THETA (0,1.46818) FIX ; 16. DIS_i4

$THETA -0.998269 FIX ; 17. DIF1_i4

$THETA (0,1.77607,1000000) FIX ; 18. DIF2_i4

$THETA (0,1.60299,1000000) FIX ; 19. DIF3_i4

$THETA (0,1.69139,1000000) FIX ; 20. DIF4_i4

$THETA (0,2.32082) FIX ; 21. DIS_i5

$THETA -0.742252 FIX ; 22. DIF1_i5

$THETA (0,1.66937,1000000) FIX ; 23. DIF2_i5

$THETA (0,1.84594,1000000) FIX ; 24. DIF3_i5

$THETA (0,1.82699,1000000) FIX ; 25. DIF4_i5

$THETA (0,2.20189) FIX ; 26. DIS_i6

$THETA -0.705819 FIX ; 27. DIF1_i6

$THETA (0,1.64988,1000000) FIX ; 28. DIF2_i6

$THETA (0,1.85796,1000000) FIX ; 29. DIF3_i6

$THETA (0,1.92845,1000000) FIX ; 30. DIF4_i6

$THETA (0,2.64433) FIX ; 31. DIS_i7

$THETA -1.60374 FIX ; 32. DIF1_i7

$THETA (0,1.70428,1000000) FIX ; 33. DIF2_i7

$THETA (0,1.83088,1000000) FIX ; 34. DIF3_i7

$THETA (0,1.59472,1000000) FIX ; 35. DIF4_i7

$THETA (0,1.47392) FIX ; 36. DIS_i8

$THETA -1.91304 FIX ; 37. DIF1_i8

$THETA (0,1.9511,1000000) FIX ; 38. DIF2_i8

$THETA (0,2.05401,1000000) FIX ; 39. DIF3_i8

$THETA 0 FIX ; 40. DIF4_i8

$THETA (0,2.05372) FIX ; 41. DIS_i9

$THETA -0.645367 FIX ; 42. DIF1_i9

$THETA (0,1.48577,1000000) FIX ; 43. DIF2_i9

$THETA (0,1.50762,1000000) FIX ; 44. DIF3_i9

$THETA (0,1.75968,1000000) FIX ; 45. DIF4_i9

$THETA (0,2.58414) FIX ; 46. DIS_i10

$THETA -1.16306 FIX ; 47. DIF1_i10

$THETA (0,1.51045,1000000) FIX ; 48. DIF2_i10

$THETA (0,1.63669,1000000) FIX ; 49. DIF3_i10

$THETA 0 FIX ; 50. DIF4_i10

$THETA (0,2.16307) FIX ; 51. DIS_i11

$THETA -1.64542 FIX ; 52. DIF1_i11

$THETA (0,1.45046,1000000) FIX ; 53. DIF2_i11

$THETA (0,1.70627,1000000) FIX ; 54. DIF3_i11

$THETA 0 FIX ; 55. DIF4_i11

$THETA (0,1.96895) FIX ; 56. DIS_i12

$THETA -1.30254 FIX ; 57. DIF1_i12

$THETA (0,1.98965,1000000) FIX ; 58. DIF2_i12

$THETA (0,1.83572,1000000) FIX ; 59. DIF3_i12

$THETA (0,1.70908,1000000) FIX ; 60. DIF4_i12

$THETA (0,1.3071) FIX ; 61. DIS_i13

$THETA -0.343326 FIX ; 62. DIF1_i13

$THETA (0,1.8725,1000000) FIX ; 63. DIF2_i13

$THETA (0,2.00766,1000000) FIX ; 64. DIF3_i13

$THETA (0,1.91018,1000000) FIX ; 65. DIF4_i13

$THETA (0,1.46874) FIX ; 66. DIS_i14

$THETA -0.502047 FIX ; 67. DIF1_i14

$THETA (0,1.72087,1000000) FIX ; 68. DIF2_i14

$THETA (0,1.91192,1000000) FIX ; 69. DIF3_i14

$THETA 0 FIX ; 70. DIF4_i14

$THETA 0 FIX ; 71. baseline time==0

$THETA -0.338473 ; 72. baseline time!=0

$OMEGA 1 FIX ; 1. iiv_baseline time==0

$OMEGA 1.60876 ; 2. ivv_baseline time !=0

$ESTIMATION MAXEVAL=9999 METHOD=1 LAPLACE LIKE PRINT=1 NSIG=2 NOABORT MSFO=msf2_updated

$COVARIANCE PRINT=E


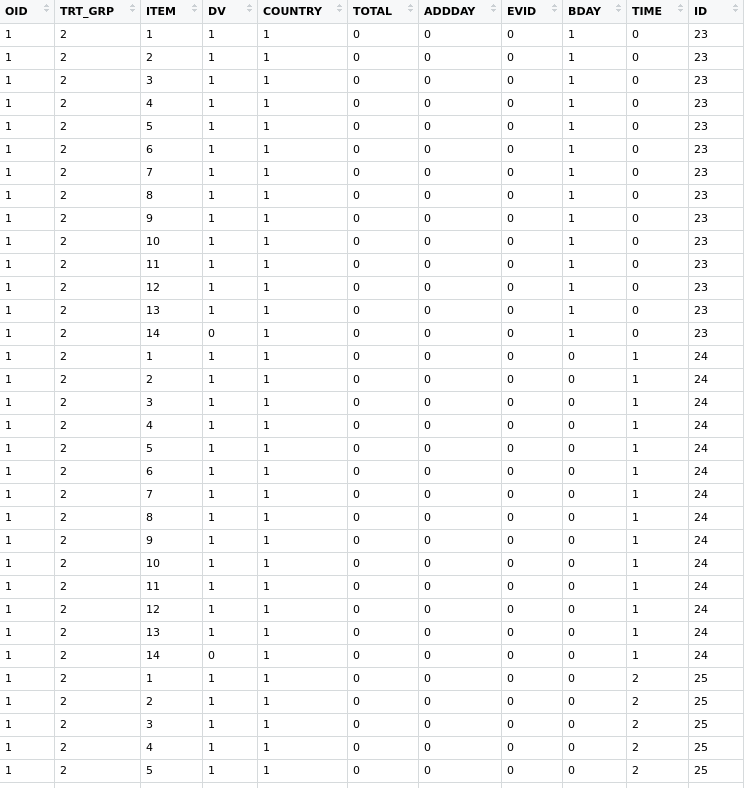


**NONMEM control stream for the intermediate step with ICF parameters fixed from step 1 and a large variance for θ to obtain θ_i_ used as DV in step 2**

$SIZES MAXFCN=100000000 NO=25000

$PROBLEM COPD PRO data modelling: ICC parameters

$INPUT OID TRT_GRP ITEM DV COUNTRY TOTAL ADDDAY EVID BDAY TIME ID

; time=newday-1, so that time starts at 0

; (new day is same as day, except for ADDDAY rows, see below)

; items 3, 8, 10, 11, 14 have DV 0-3; and the rest 0-4

; OID is the original ID number

; total is a flag indicating that item 99 is total score

; add day is a flag indicating that a day was added into the study (due to missing data); DV is -99 then

; BDAY is baseline time (BDAY=1 for positive time values, BDAY=2 for negative time values (screening period) BDAY=0 no baseline values).

; Most patients start at day 1, but some don’t so BDAY is a flag to indicate the first day of treatment for each patient (baseline)

; ID is a new ID at each time point (Independent occasion approach for ICC estimates)

$DATA study2_bytime_20200707.csv IGNORE=@

IGNORE=(ADDDAY==1); ignore days that were added in

IGNORE=(TOTAL==1); ignore total score rows

IGNORE=(EVID==4); no MM elements included

IGNORE=(TIME<-14); baseline is calculated from day -14

IGNORE(ITEM.EQ.12); item does not belong to E-RS:COPD

IGNORE(ITEM.EQ.13); item does not belong to E-RS:COPD

IGNORE(ITEM.EQ.14); item does not belong to E-RS:COPD

$PRED

; --------- (re)set the PDV --------------------------

IF(NEWIND.NE.2.OR.BDAY.EQ.1) THEN ; first row of a subject or an item

IF(EVID==0) TMPDV=DV ; first PDV=DV

NREC=1

OLDTIME=TIME

ENDIF

XNRC = NREC

IF(TIME>OLDTIME) NREC=NREC+1

IF(EVID==0) PDV=TMPDV

;------------ item parameter selection ----------------------

IF(ITEM==1) THEN

DIS = THETA(1)

DIF1 = THETA(2)

DIF2 = THETA(3)

DIF3 = THETA(4)

DIF4 = THETA(5)

ENDIF

IF(ITEM==2) THEN

DIS = THETA(6)

DIF1 = THETA(7)

DIF2 = THETA(8)

DIF3 = THETA(9)

DIF4 = THETA(10)

ENDIF

IF(ITEM==3) THEN

DIS = THETA(11)

DIF1 = THETA(12)

DIF2 = THETA(13)

DIF3 = THETA(14)

DIF4 = THETA(15) ; no need, only 0-3

ENDIF

IF(ITEM==4) THEN

DIS = THETA(16)

DIF1 = THETA(17)

DIF2 = THETA(18)

DIF3 = THETA(19)

DIF4 = THETA(20)

ENDIF

IF(ITEM==5) THEN

DIS = THETA(21)

DIF1 = THETA(22)

DIF2 = THETA(23)

DIF3 = THETA(24)

DIF4 = THETA(25)

ENDIF

IF(ITEM==6) THEN

DIS = THETA(26)

DIF1 = THETA(27)

DIF2 = THETA(28)

DIF3 = THETA(29)

DIF4 = THETA(30)

ENDIF

IF(ITEM==7) THEN

DIS = THETA(31)

DIF1 = THETA(32)

DIF2 = THETA(33)

DIF3 = THETA(34)

DIF4 = THETA(35)

ENDIF

IF(ITEM==8) THEN

DIS = THETA(36)

DIF1 = THETA(37)

DIF2 = THETA(38)

DIF3 = THETA(39)

DIF4 = THETA(40) ; no need, only 0-3

ENDIF

IF(ITEM==9) THEN

DIS = THETA(41)

DIF1 = THETA(42)

DIF2 = THETA(43)

DIF3 = THETA(44)

DIF4 = THETA(45)

ENDIF

IF(ITEM==10) THEN

DIS = THETA(46)

DIF1 = THETA(47)

DIF2 = THETA(48)

DIF3 = THETA(49)

DIF4 = THETA(50) ; no need, only 0-3

ENDIF

IF(ITEM==11) THEN

DIS = THETA(51)

DIF1 = THETA(52)

DIF2 = THETA(53)

DIF3 = THETA(54)

DIF4 = THETA(55) ; no need, only 0-3

ENDIF

IF(ITEM==12) THEN

DIS = THETA(56)

DIF1 = THETA(57)

DIF2 = THETA(58)

DIF3 = THETA(59)

DIF4 = THETA(60)

ENDIF

IF(ITEM==13) THEN

DIS = THETA(61)

DIF1 = THETA(62)

DIF2 = THETA(63)

DIF3 = THETA(64)

DIF4 = THETA(65)

ENDIF

IF(ITEM==14) THEN

DIS = THETA(66)

DIF1 = THETA(67)

DIF2 = THETA(68)

DIF3 = THETA(69)

DIF4 = THETA(70) ; no need, only 0-3

ENDIF

;----------------- hidden variable model -------------------

MU_1 = THETA(71)

BASELINE = MU_1 + ETA(1)

PSI=BASELINE ; COPD disease severity

;------ 2 parameter logit model implementation (4/5 states) ------

;------ constrain different states to be >= than the previous -------

DIFS1 = DIF1

DIFS2 = DIFS1+DIF2

DIFS3 = DIFS2+DIF3

DIFS4 = DIFS3+DIF4

IF(ITEM==3.OR.ITEM==8.OR.ITEM==10.OR.ITEM==11.OR.ITEM==14) DIFS4 = 0

; ------ probabilities for Y greater than 1 etc -------------

PGE1 = EXP(DIS*(PSI-DIFS1))/(1+EXP(DIS*(PSI-DIFS1)))

PGE2 = EXP(DIS*(PSI-DIFS2))/(1+EXP(DIS*(PSI-DIFS2)))

PGE3 = EXP(DIS*(PSI-DIFS3))/(1+EXP(DIS*(PSI-DIFS3)))

PGE4 = EXP(DIS*(PSI-DIFS4))/(1+EXP(DIS*(PSI-DIFS4)))

IF(ITEM==3.OR.ITEM==8.OR.ITEM==10.OR.ITEM==11.OR.ITEM==14) PGE4 = 0

; ------ probabilities for Y=0 etc --------------------------

P0 = 1-PGE1

P1 = PGE1-PGE2

P2 = PGE2-PGE3

P3 = PGE3-PGE4

IF(ITEM==3.OR.ITEM==8.OR.ITEM==10.OR.ITEM==11.OR.ITEM==14) P3 = PGE3

P4 = PGE4

IF(ITEM==3.OR.ITEM==8.OR.ITEM==10.OR.ITEM==11.OR.ITEM==14) P4 = 0

IF(DV==0) Y=P0

IF(DV==1) Y=P1

IF(DV==2) Y=P2

IF(DV==3) Y=P3

IF(DV==4) Y=P4

IPRED = (P0*0)+(P1*1)+(P2*2)+(P3*3)+(P4*4)

RES = DV - IPRED

;----- get XDV ------------------------------------

XDV=0

IF (EVID==0) XDV = PDV*10 + DV

;----- remember TIME, DV --------------------------

OLDTIME=TIME

IF(EVID==0.AND.ITEM.NE.99) TMPDV = DV

$THETA (0,1.79211) FIX ; 1. DIS_i1

$THETA -1.03362 FIX ; 2. DIF1_i1

$THETA (0,2.03489,1000000) FIX ; 3. DIF2_i1

$THETA (0,1.95689,1000000) FIX ; 4. DIF3_i1

$THETA (0,1.98008,1000000) FIX ; 5. DIF4_i1

$THETA (0,1.13922) FIX ; 6. DIS_i2

$THETA -2.29355 FIX ; 7. DIF1_i2

$THETA (0,2.74145,1000000) FIX ; 8. DIF2_i2

$THETA (0,2.53703,1000000) FIX ; 9. DIF3_i2

$THETA (0,2.74127,1000000) FIX ; 10. DIF4_i2

$THETA (0,1.10396) FIX ; 11. DIS_i3

$THETA -1.91627 FIX ; 12. DIF1_i3

$THETA (0,5.75838,1000000) FIX ; 13. DIF2_i3

$THETA (0,2.96354,1000000) FIX ; 14. DIF3_i3

$THETA 0 FIX ; 15. DIF4_i3

$THETA (0,1.46818) FIX ; 16. DIS_i4

$THETA -0.998269 FIX ; 17. DIF1_i4

$THETA (0,1.77607,1000000) FIX ; 18. DIF2_i4

$THETA (0,1.60299,1000000) FIX ; 19. DIF3_i4

$THETA (0,1.69139,1000000) FIX ; 20. DIF4_i4

$THETA (0,2.32082) FIX ; 21. DIS_i5

$THETA -0.742252 FIX ; 22. DIF1_i5

$THETA (0,1.66937,1000000) FIX ; 23. DIF2_i5

$THETA (0,1.84594,1000000) FIX ; 24. DIF3_i5

$THETA (0,1.82699,1000000) FIX ; 25. DIF4_i5

$THETA (0,2.20189) FIX ; 26. DIS_i6

$THETA -0.705819 FIX ; 27. DIF1_i6

$THETA (0,1.64988,1000000) FIX ; 28. DIF2_i6

$THETA (0,1.85796,1000000) FIX ; 29. DIF3_i6

$THETA (0,1.92845,1000000) FIX ; 30. DIF4_i6

$THETA (0,2.64433) FIX ; 31. DIS_i7

$THETA -1.60374 FIX ; 32. DIF1_i7

$THETA (0,1.70428,1000000) FIX ; 33. DIF2_i7

$THETA (0,1.83088,1000000) FIX ; 34. DIF3_i7

$THETA (0,1.59472,1000000) FIX ; 35. DIF4_i7

$THETA (0,1.47392) FIX ; 36. DIS_i8

$THETA -1.91304 FIX ; 37. DIF1_i8

$THETA (0,1.9511,1000000) FIX ; 38. DIF2_i8

$THETA (0,2.05401,1000000) FIX ; 39. DIF3_i8

$THETA 0 FIX ; 40. DIF4_i8

$THETA (0,2.05372) FIX ; 41. DIS_i9

$THETA -0.645367 FIX ; 42. DIF1_i9

$THETA (0,1.48577,1000000) FIX ; 43. DIF2_i9

$THETA (0,1.50762,1000000) FIX ; 44. DIF3_i9

$THETA (0,1.75968,1000000) FIX ; 45. DIF4_i9

$THETA (0,2.58414) FIX ; 46. DIS_i10

$THETA -1.16306 FIX ; 47. DIF1_i10

$THETA (0,1.51045,1000000) FIX ; 48. DIF2_i10

$THETA (0,1.63669,1000000) FIX ; 49. DIF3_i10

$THETA 0 FIX ; 50. DIF4_i10

$THETA (0,2.16307) FIX ; 51. DIS_i11

$THETA -1.64542 FIX ; 52. DIF1_i11

$THETA (0,1.45046,1000000) FIX ; 53. DIF2_i11

$THETA (0,1.70627,1000000) FIX ; 54. DIF3_i11

$THETA 0 FIX ; 55. DIF4_i11

$THETA (0,1.96895) FIX ; 56. DIS_i12

$THETA -1.30254 FIX ; 57. DIF1_i12

$THETA (0,1.98965,1000000) FIX ; 58. DIF2_i12

$THETA (0,1.83572,1000000) FIX ; 59. DIF3_i12

$THETA (0,1.70908,1000000) FIX ; 60. DIF4_i12

$THETA (0,1.3071) FIX ; 61. DIS_i13

$THETA -0.343326 FIX ; 62. DIF1_i13

$THETA (0,1.8725,1000000) FIX ; 63. DIF2_i13

$THETA (0,2.00766,1000000) FIX ; 64. DIF3_i13

$THETA (0,1.91018,1000000) FIX ; 65. DIF4_i13

$THETA (0,1.46874) FIX ; 66. DIS_i14

$THETA -0.502047 FIX ; 67. DIF1_i14

$THETA (0,1.72087,1000000) FIX ; 68. DIF2_i14

$THETA (0,1.91192,1000000) FIX ; 69. DIF3_i14

$THETA 0 FIX ; 70. DIF4_i14

$THETA -0.319557 ; 71. baseline (E-RS:COPD-11 items)

$OMEGA 25 ; 1. iiv_baseline

$ESTIMATION METHOD=SAEM LAPLACE LIKE EONLY=1 PRINT=10 NBURN=200

NITER=0

$COVARIANCE UNCONDITIONAL PRINT=E

$TABLE OID ID TIME TRT_GRP FL_SMOK COUNTRY2 TOTAL_PRO TOTAL_RS

BDAY PSI ETAS(1:LAST) NOPRINT ONEHEADER VARCALC=1

FILE=tab128_var25 FORMAT=s1PE23.16

**NONMEM control stream and snippet of the data for the longitudinal model (step 2)**

$SIZES MAXFCN=100000000 NO=25000 DIMNEW=2000

$PROBLEM COPD PRO data modelling: longitudinal model – modelling iPSI

$ABBREVIATED PROTECT

$INPUT ID TIME TRT_GRP FL_SMOK COUNTRY2 TOTAL_PRO TOTAL_RS

BDAY BASEM DV PSI_VAR

; ID: original ID

; time:newday-1, so that time starts at 0

; TRT_GRP: treatment group (TRT_GRP==1 FF/UMEC/VI; TRT_GRP==2 BUD/FOR)

; FL_SMOK: smoking covariate (FL_SMOK==1 smokers; FL_SMOK==2 non-smokers)

; country2: country

; total_pro: total EXACT score (0 to 100)

; total_rs: sumscore is a sum of item (1 to 11) sub-scores (0 to 40)

; BDAY: baseline day

; BASEM: flag for IDs with missing baseline value (BASEM=1) defined as the ; mean total score value during baseline period defined as from day -14 to ; day -1. Baseline was only calculated if 7 or more days had non-missing ; score present as original MMRM analysis did.

; DV: PSI values from run128_var25.phi (intermediate step)

; PSI_VAR: PSI uncertainty from run128_var25.phi (intermediate step)

$DATA study2_PHIrun128_var25.csv

IGNORE=@

IGNORE(TIME>168); MMRM includes only data up to week 24

IGNORE(BASEM.EQ.1); ignore individuals with missing baseline

$PRED

;----------- for stratified VPC --------------------

STRT=0

IF(TRT_GRP==1) STRT=1

IF(TRT_GRP==2) STRT=2

;----------- Longitudinal model --------------------

TIMEY = (TIME/365)

IF(TIMEY.LT.0) TIMEY = 0

;Smoking effect

;FL_SMOK=1 smoking

;FL_SMOK=2 no smoking (most common)

IF(FL_SMOK.EQ.2) SMOKCOV = 0

IF(FL_SMOK.EQ.1) SMOKCOV = THETA(11)

;Geographical region effect

;Country

;Greece = 1

;Bulgaria= 2

;Czech Republic =3

;Estonia = 4

;Germany =5

;Hungary =6

;Italy = 7

;Poland = 8

;Romania = 9

;Russian Federation = 10

;Slovakia = 11

;Ukraine =12

;China = 13

;Korea = 14

;Republic of Mexico = 15

;Europe 1 (21%): Germany, Greece, Italy

;Europe 2 (24%): Russian Federation,Ukraine

;Europe 3 (21%): Bulgaria, Hungary, Romania, Slovakia

;Europe 4 (18%): Czech Republic, Estonia, Poland

;Asia (6%): China, Republic of Korea

;Mexico (10%)

REGION1=0

REGION3=0

REGION4=0

REGION5=0

REGION6=0

IF(COUNTRY2.EQ.5.OR.COUNTRY2.EQ.1.OR.COUNTRY2.EQ.7) REGION1 = THETA(12)

IF(COUNTRY2.EQ.2.OR.COUNTRY2.EQ.6.OR.COUNTRY2.EQ.9.OR.COUNTRY2.EQ.11) REGION3 = THETA(13)

IF(COUNTRY2.EQ.3.OR.COUNTRY2.EQ.4.OR.COUNTRY2.EQ.8) REGION4 = THETA(14)

IF(COUNTRY2.EQ.13.OR.COUNTRY2.EQ.14) REGION5 = THETA(15)

IF(COUNTRY2.EQ.15) REGION6 = THETA(16)

REGIONCOV = REGION1 + REGION3 + REGION4 + REGION5 + REGION6

;---- Parameters stratified by arms ----

;TRT_GRP=1 FF/UMEC/VI

;TRT_GRP=2 BUD/FOR

IF(TRT_GRP.EQ.1) TVBASE = THETA(1) + SMOKCOV + REGIONCOV

IF(TRT_GRP.EQ.1) BASELINE = TVBASE + ETA(4)

IF(TRT_GRP.EQ.2) TVBASE = THETA(6) + SMOKCOV + REGIONCOV

IF(TRT_GRP.EQ.2) BASELINE = TVBASE + ETA(7)

IF(TRT_GRP.EQ.1) TVTPROG = THETA(2)

IF(TRT_GRP.EQ.1) TPROG = TVTPROG *EXP(ETA(1))

IF(TRT_GRP.EQ.2) TVTPROG = THETA(7)

IF(TRT_GRP.EQ.2) TPROG = TVTPROG *EXP(ETA(5))

IF(TRT_GRP.EQ.1) TVPMAX = THETA(3)

IF(TRT_GRP.EQ.1) PMAX = TVPMAX + ETA(2)

IF(TRT_GRP.EQ.2) TVPMAX = THETA(8)

IF(TRT_GRP.EQ.2) PMAX = TVPMAX + ETA(6)

IF(TRT_GRP.EQ.1) TVHILL = THETA(4)

IF(TRT_GRP.EQ.1) HILL = TVHILL

IF(TRT_GRP.EQ.2) TVHILL = THETA(9)

IF(TRT_GRP.EQ.2) HILL = TVHILL

;---- Offset effect ----

OFFSET = 0

IF(TRT_GRP.EQ.1.AND.TIMEY.GT.0) TVOFFSET = THETA(5)

IF(TRT_GRP.EQ.1.AND.TIMEY.GT.0) OFFSET = TVOFFSET + ETA(3)

IF(TRT_GRP.EQ.2.AND.TIMEY.GT.0) TVOFFSET = THETA(10)

IF(TRT_GRP.EQ.2.AND.TIMEY.GT.0) OFFSET = TVOFFSET + ETA(8)

;---- Weibull function ----

RATED = LOG(2)/TPROG

RATEDTIME = RATED*TIMEY

DISMOD = 0

IF(TIMEY.GT.0) DISMOD = PMAX*(1-EXP(-(RATEDTIME**HILL)))

;---- Latent variable ----

PSI = BASELINE + DISMOD + OFFSET

IPRED = PSI ; iPSI from run128_var25.phi

W = SQRT(PSI_VAR) ; SE of iPSI from run128_var25.phi

Y = IPRED + W * EPS(1) + EPS(2)

;--------- Relationship between total score and PSI ERS (11 ITEMS) -----

IF(PSI.LE.-8.775543892) TS = 0.000942760942761

IF(PSI.GT.-8.775543892.AND.PSI.LE.-8.332094007) TS= 0.002087542087542

IF(PSI.GT.-8.332094007.AND.PSI.LE.-7.878533751) TS= 0.003838383838384

IF(PSI.GT.-7.878533751.AND.PSI.LE.-7.434058916) TS= 0.005656565656566

IF(PSI.GT.-7.434058916.AND.PSI.LE.-6.984862431) TS= 0.00976430976431

IF(PSI.GT.-6.984862431.AND.PSI.LE.-6.536552855) TS= 0.017441077441077

IF(PSI.GT.-6.536552855.AND.PSI.LE.-6.083757239) TS= 0.023973063973064

IF(PSI.GT.-6.083757239.AND.PSI.LE.-5.629505724) TS= 0.046734006734007

IF(PSI.GT.-5.629505724.AND.PSI.LE.-5.173188061) TS= 0.08026936026936

IF(PSI.GT.-5.173188061.AND.PSI.LE.-4.718352835) TS= 0.127003367003367

IF(PSI.GT.-4.718352835.AND.PSI.LE.-4.265694781) TS= 0.232592592592593

IF(PSI.GT.-4.265694781.AND.PSI.LE.-3.962071535) TS= 0.310841750841751

IF(PSI.GT.-3.962071535.AND.PSI.LE.-3.867145118) TS= 0.347811447811448

IF(PSI.GT.-3.867145118.AND.PSI.LE.-3.772459212) TS= 0.380942760942761

IF(PSI.GT.-3.772459212.AND.PSI.LE.-3.677247892) TS= 0.432323232323232

IF(PSI.GT.-3.677247892.AND.PSI.LE.-3.584014034) TS= 0.468282828282828

IF(PSI.GT.-3.584014034.AND.PSI.LE.-3.489947219) TS= 0.548148148148148

IF(PSI.GT.-3.489947219.AND.PSI.LE.-3.395964162) TS= 0.587542087542088

IF(PSI.GT.-3.395964162.AND.PSI.LE.-3.302082350) TS= 0.661346801346801

IF(PSI.GT.-3.302082350.AND.PSI.LE.-3.207769293) TS= 0.718653198653199

IF(PSI.GT.-3.207769293.AND.PSI.LE.-3.113916545) TS= 0.821750841750842

IF(PSI.GT.-3.113916545.AND.PSI.LE.-3.019800061) TS= 0.919057239057239

IF(PSI.GT.-3.019800061.AND.PSI.LE.-2.926450290) TS= 1.00969696969697

IF(PSI.GT.-2.926450290.AND.PSI.LE.-2.835353125) TS= 1.11912457912458

IF(PSI.GT.-2.835353125.AND.PSI.LE.-2.745258734) TS= 1.25313131313131

IF(PSI.GT.-2.745258734.AND.PSI.LE.-2.655348936) TS= 1.37198653198653

IF(PSI.GT.-2.655348936.AND.PSI.LE.-2.567411785) TS= 1.52565656565657

IF(PSI.GT.-2.567411785.AND.PSI.LE.-2.481035751) TS= 1.66680134680135

IF(PSI.GT.-2.481035751.AND.PSI.LE.-2.396683805) TS= 1.86606060606061

IF(PSI.GT.-2.396683805.AND.PSI.LE.-2.315625535) TS= 2.0156228956229

IF(PSI.GT.-2.315625535.AND.PSI.LE.-2.235988882) TS= 2.19925925925926

IF(PSI.GT.-2.235988882.AND.PSI.LE.-2.159054795) TS= 2.40228956228956

IF(PSI.GT.-2.159054795.AND.PSI.LE.-2.085839387) TS= 2.6226936026936

IF(PSI.GT.-2.085839387.AND.PSI.LE.-2.014283199) TS= 2.83010101010101

IF(PSI.GT.-2.014283199.AND.PSI.LE.-1.944122027) TS= 3.06276094276094

IF(PSI.GT.-1.944122027.AND.PSI.LE.-1.876710088) TS= 3.29063973063973

IF(PSI.GT.-1.876710088.AND.PSI.LE.-1.811653414) TS= 3.50915824915825

IF(PSI.GT.-1.811653414.AND.PSI.LE.-1.749357051) TS= 3.76141414141414

IF(PSI.GT.-1.749357051.AND.PSI.LE.-1.689669441) TS= 4.02430976430976

IF(PSI.GT.-1.689669441.AND.PSI.LE.-1.631739751) TS= 4.22377104377104

IF(PSI.GT.-1.631739751.AND.PSI.LE.-1.575946431) TS= 4.49717171717172

IF(PSI.GT.-1.575946431.AND.PSI.LE.-1.521647387) TS= 4.7186531986532

IF(PSI.GT.-1.521647387.AND.PSI.LE.-1.469366552) TS= 4.97582491582492

IF(PSI.GT.-1.469366552.AND.PSI.LE.-1.419502694) TS= 5.23508417508418

IF(PSI.GT.-1.419502694.AND.PSI.LE.-1.370939347) TS= 5.45104377104377

IF(PSI.GT.-1.370939347.AND.PSI.LE.-1.323737131) TS= 5.67616161616162

IF(PSI.GT.-1.323737131.AND.PSI.LE.-1.277947973) TS= 5.91077441077441

IF(PSI.GT.-1.277947973.AND.PSI.LE.-1.233491717) TS= 6.11993265993266

IF(PSI.GT.-1.233491717.AND.PSI.LE.-1.190275448) TS= 6.33858585858586

IF(PSI.GT.-1.190275448.AND.PSI.LE.-1.148105886) TS= 6.58949494949495

IF(PSI.GT.-1.148105886.AND.PSI.LE.-1.106792000) TS= 6.79090909090909

IF(PSI.GT.-1.106792000.AND.PSI.LE.-1.067530027) TS= 7.02181818181818

IF(PSI.GT.-1.067530027.AND.PSI.LE.-1.029065811) TS= 7.22141414141414

IF(PSI.GT.-1.029065811.AND.PSI.LE.-0.990998673) TS= 7.44175084175084

IF(PSI.GT.-0.990998673.AND.PSI.LE.-0.954145978) TS= 7.60154882154882

IF(PSI.GT.-0.954145978.AND.PSI.LE.-0.917849541) TS= 7.81131313131313

IF(PSI.GT.-0.917849541.AND.PSI.LE.-0.882258560) TS= 8.02619528619529

IF(PSI.GT.-0.882258560.AND.PSI.LE.-0.847456873) TS= 8.20329966329966

IF(PSI.GT.-0.847456873.AND.PSI.LE.-0.813765597) TS= 8.37461279461279

IF(PSI.GT.-0.813765597.AND.PSI.LE.-0.780315861) TS= 8.57690235690236

IF(PSI.GT.-0.780315861.AND.PSI.LE.-0.747235280) TS= 8.74734006734007

IF(PSI.GT.-0.747235280.AND.PSI.LE.-0.715070552) TS= 8.92754208754209

IF(PSI.GT.-0.715070552.AND.PSI.LE.-0.683480819) TS= 9.0986531986532

IF(PSI.GT.-0.683480819.AND.PSI.LE.-0.652104206) TS= 9.29434343434343

IF(PSI.GT.-0.652104206.AND.PSI.LE.-0.621162782) TS= 9.43070707070707

IF(PSI.GT.-0.621162782.AND.PSI.LE.-0.590566032) TS= 9.61326599326599

IF(PSI.GT.-0.590566032.AND.PSI.LE.-0.560588794) TS= 9.80127946127946

IF(PSI.GT.-0.560588794.AND.PSI.LE.-0.530489864) TS= 9.95710437710438

IF(PSI.GT.-0.530489864.AND.PSI.LE.-0.500767069) TS= 10.1215488215488

IF(PSI.GT.-0.500767069.AND.PSI.LE.-0.471524842) TS= 10.2909090909091

IF(PSI.GT.-0.471524842.AND.PSI.LE.-0.442599263) TS= 10.4455892255892

IF(PSI.GT.-0.442599263.AND.PSI.LE.-0.413829504) TS= 10.6290235690236

IF(PSI.GT.-0.413829504.AND.PSI.LE.-0.385232507) TS= 10.7985185185185

IF(PSI.GT.-0.385232507.AND.PSI.LE.-0.356762737) TS= 10.9544781144781

IF(PSI.GT.-0.356762737.AND.PSI.LE.-0.328979723) TS= 11.0814814814815

IF(PSI.GT.-0.328979723.AND.PSI.LE.-0.301098593) TS= 11.2457239057239

IF(PSI.GT.-0.301098593.AND.PSI.LE.-0.273412057) TS= 11.3670707070707

IF(PSI.GT.-0.273412057.AND.PSI.LE.-0.245688145) TS= 11.5621548821549

IF(PSI.GT.-0.245688145.AND.PSI.LE.-0.218095107) TS= 11.6993939393939

IF(PSI.GT.-0.218095107.AND.PSI.LE.-0.190865333) TS= 11.8723905723906

IF(PSI.GT.-0.190865333.AND.PSI.LE.-0.163583949) TS= 12.0426262626263

IF(PSI.GT.-0.163583949.AND.PSI.LE.-0.136415440) TS= 12.1650505050505

IF(PSI.GT.-0.136415440.AND.PSI.LE.-0.109337123) TS= 12.3101683501684

IF(PSI.GT.-0.109337123.AND.PSI.LE.-0.082393159) TS= 12.4693602693603

IF(PSI.GT.-0.082393159.AND.PSI.LE.-0.055405015) TS= 12.6509764309764

IF(PSI.GT.-0.055405015.AND.PSI.LE.-0.028483575) TS= 12.7683501683502

IF(PSI.GT.-0.028483575.AND.PSI.LE.-0.001814754) TS= 12.9480134680135

IF(PSI.GT.-0.001814754.AND.PSI.LE.0.024936777) TS= 13.1251851851852

IF(PSI.GT.0.024936777.AND.PSI.LE.0.051775202) TS= 13.260404040404

IF(PSI.GT.0.051775202.AND.PSI.LE.0.078688175) TS= 13.3980471380471

IF(PSI.GT.0.078688175.AND.PSI.LE.0.105564760) TS= 13.5651178451178

IF(PSI.GT.0.105564760.AND.PSI.LE.0.132682927) TS= 13.6955555555556

IF(PSI.GT.0.132682927.AND.PSI.LE.0.159841209) TS= 13.8762962962963

IF(PSI.GT.0.159841209.AND.PSI.LE.0.186999125) TS= 14.0211447811448

IF(PSI.GT.0.186999125.AND.PSI.LE.0.214491554) TS= 14.1481481481481

IF(PSI.GT.0.214491554.AND.PSI.LE.0.241998999) TS= 14.3230976430976

IF(PSI.GT.0.241998999.AND.PSI.LE.0.269393076) TS= 14.4645117845118

IF(PSI.GT.0.269393076.AND.PSI.LE.0.296960773) TS= 14.6614141414141

IF(PSI.GT.0.296960773.AND.PSI.LE.0.324597850) TS= 14.7911784511785

IF(PSI.GT.0.324597850.AND.PSI.LE.0.352810982) TS= 14.9816161616162

IF(PSI.GT.0.352810982.AND.PSI.LE.0.381024222) TS= 15.0991919191919

IF(PSI.GT.0.381024222.AND.PSI.LE.0.409808783) TS= 15.2808754208754

IF(PSI.GT.0.409808783.AND.PSI.LE.0.438837277) TS= 15.4616835016835

IF(PSI.GT.0.438837277.AND.PSI.LE.0.467766535) TS= 15.623164983165

IF(PSI.GT.0.467766535.AND.PSI.LE.0.497191593) TS= 15.7718518518519

IF(PSI.GT.0.497191593.AND.PSI.LE.0.526845734) TS= 15.9430303030303

IF(PSI.GT.0.526845734.AND.PSI.LE.0.556388971) TS= 16.1047138047138

IF(PSI.GT.0.556388971.AND.PSI.LE.0.586462774) TS= 16.2890909090909

IF(PSI.GT.0.586462774.AND.PSI.LE.0.616588817) TS= 16.4760269360269

IF(PSI.GT.0.616588817.AND.PSI.LE.0.647749884) TS= 16.6614814814815

IF(PSI.GT.0.647749884.AND.PSI.LE.0.679160288) TS= 16.7977777777778

IF(PSI.GT.0.679160288.AND.PSI.LE.0.710868139) TS= 17.0026262626263

IF(PSI.GT.0.710868139.AND.PSI.LE.0.743043042) TS= 17.1602693602694

IF(PSI.GT.0.743043042.AND.PSI.LE.0.775749526) TS= 17.3591919191919

IF(PSI.GT.0.775749526.AND.PSI.LE.0.808694438) TS= 17.5540740740741

IF(PSI.GT.0.808694438.AND.PSI.LE.0.842503931) TS= 17.7412794612795

IF(PSI.GT.0.842503931.AND.PSI.LE.0.877024357) TS= 17.933063973064

IF(PSI.GT.0.877024357.AND.PSI.LE.0.911967207) TS= 18.1200673400673

IF(PSI.GT.0.911967207.AND.PSI.LE.0.947740365) TS= 18.3186531986532

IF(PSI.GT.0.947740365.AND.PSI.LE.0.983989702) TS= 18.4849831649832

IF(PSI.GT.0.983989702.AND.PSI.LE.1.021506505) TS= 18.7447811447811

IF(PSI.GT.1.021506505.AND.PSI.LE.1.059921933) TS= 18.9523905723906

IF(PSI.GT.1.059921933.AND.PSI.LE.1.099630848) TS= 19.150303030303

IF(PSI.GT.1.099630848.AND.PSI.LE.1.140043832) TS= 19.3750168350168

IF(PSI.GT.1.140043832.AND.PSI.LE.1.181319549) TS= 19.603771043771

IF(PSI.GT.1.181319549.AND.PSI.LE.1.224314815) TS= 19.8353535353535

IF(PSI.GT.1.224314815.AND.PSI.LE.1.268651185) TS= 20.0828282828283

IF(PSI.GT.1.268651185.AND.PSI.LE.1.314374404) TS= 20.3350168350168

IF(PSI.GT.1.314374404.AND.PSI.LE.1.361268074) TS= 20.5938047138047

IF(PSI.GT.1.361268074.AND.PSI.LE.1.409210512) TS= 20.8346127946128

IF(PSI.GT.1.409210512.AND.PSI.LE.1.458784020) TS= 21.1381818181818

IF(PSI.GT.1.458784020.AND.PSI.LE.1.510588552) TS= 21.3716498316498

IF(PSI.GT.1.510588552.AND.PSI.LE.1.564418936) TS= 21.683771043771

IF(PSI.GT.1.564418936.AND.PSI.LE.1.619888519) TS= 21.9704377104377

IF(PSI.GT.1.619888519.AND.PSI.LE.1.677090687) TS= 22.3049158249158

IF(PSI.GT.1.677090687.AND.PSI.LE.1.737555320) TS= 22.6344107744108

IF(PSI.GT.1.737555320.AND.PSI.LE.1.799400997) TS= 22.9731313131313

IF(PSI.GT.1.799400997.AND.PSI.LE.1.863578397) TS= 23.3005387205387

IF(PSI.GT.1.863578397.AND.PSI.LE.1.930899024) TS= 23.7047811447811

IF(PSI.GT.1.930899024.AND.PSI.LE.1.999643771) TS= 24.036835016835

IF(PSI.GT.1.999643771.AND.PSI.LE.2.070960640) TS= 24.4220202020202

IF(PSI.GT.2.070960640.AND.PSI.LE.2.145231401) TS= 24.8285521885522

IF(PSI.GT.2.145231401.AND.PSI.LE.2.221722754) TS= 25.2573063973064

IF(PSI.GT.2.221722754.AND.PSI.LE.2.300832667) TS= 25.6402693602694

IF(PSI.GT.2.300832667.AND.PSI.LE.2.381464626) TS= 26.0816161616162

IF(PSI.GT.2.381464626.AND.PSI.LE.2.464731414) TS= 26.5489562289562

IF(PSI.GT.2.464731414.AND.PSI.LE.2.549261448) TS= 26.919797979798

IF(PSI.GT.2.549261448.AND.PSI.LE.2.636404276) TS= 27.3738720538721

IF(PSI.GT.2.636404276.AND.PSI.LE.2.724778088) TS= 27.8003367003367

IF(PSI.GT.2.724778088.AND.PSI.LE.2.814388007) TS= 28.2340740740741

IF(PSI.GT.2.814388007.AND.PSI.LE.2.904753845) TS= 28.6708417508418

IF(PSI.GT.2.904753845.AND.PSI.LE.2.996332330) TS= 29.0653198653199

IF(PSI.GT.2.996332330.AND.PSI.LE.3.088734983) TS= 29.5189898989899

IF(PSI.GT.3.088734983.AND.PSI.LE.3.182574572) TS= 29.9135353535354

IF(PSI.GT.3.182574572.AND.PSI.LE.3.276440377) TS= 30.3240404040404

IF(PSI.GT.3.276440377.AND.PSI.LE.3.370089737) TS= 30.7230976430976

IF(PSI.GT.3.370089737.AND.PSI.LE.3.462960121) TS= 31.130303030303

IF(PSI.GT.3.462960121.AND.PSI.LE.3.556996882) TS= 31.4987878787879

IF(PSI.GT.3.556996882.AND.PSI.LE.3.651841232) TS= 31.8985858585859

IF(PSI.GT.3.651841232.AND.PSI.LE.3.746803104) TS= 32.243164983165

IF(PSI.GT.3.746803104.AND.PSI.LE.3.841729401) TS= 32.6273400673401

IF(PSI.GT.3.841729401.AND.PSI.LE.3.936248795) TS= 32.9897643097643

IF(PSI.GT.3.936248795.AND.PSI.LE.4.148309219) TS= 33.7416161616162

IF(PSI.GT.4.148309219.AND.PSI.LE.4.590940694) TS= 35.2950841750842

IF(PSI.GT.4.590940694.AND.PSI.LE.5.039508148) TS= 36.6337373737374

IF(PSI.GT.5.039508148.AND.PSI.LE.5.491795091) TS= 37.6727946127946

IF(PSI.GT.5.491795091.AND.PSI.LE.5.940894788) TS= 38.3406734006734

IF(PSI.GT.5.940894788.AND.PSI.LE.6.387543939) TS= 38.8297643097643

IF(PSI.GT.6.387543939.AND.PSI.LE.6.836367966) TS= 39.1592592592593

IF(PSI.GT.6.836367966.AND.PSI.LE.7.285584195) TS= 39.4152188552189

IF(PSI.GT.7.285584195.AND.PSI.LE.7.737254081) TS= 39.6046464646465

IF(PSI.GT.7.737254081.AND.PSI.LE.8.185744687) TS= 39.7466666666667

IF(PSI.GT.8.185744687.AND.PSI.LE.8.636998660) TS= 39.8327272727273

IF(PSI.GT.8.636998660.AND.PSI.LE.9.086867926) TS= 39.8995286195286

IF(PSI.GT.9.086867926.AND.PSI.LE.9.533969461) TS= 39.9346127946128

IF(PSI.GT.9.533969461.AND.PSI.LE.9.981423051) TS= 39.96

IF(PSI.GT.9.981423051) TS= 40

$THETA 0.327545 ; 1. baseline_T

$THETA (0.001,0.08273) ; 2. TPROG_T

$THETA -0.310089 ; 3. PMAX_T

$THETA (0.001,9.26687) ; 4. HILL_T

$THETA -0.268779 ; 5. OFFSET_T

$THETA 0.289592 ; 6. baseline_D

$THETA (0.001,0.0841009) ; 7. TPROG_D

$THETA -0.159457 ; 8. PMAX_D

$THETA (0.001,16.9738) ; 9. HILL_D

$THETA -0.0811577 ; 10. OFFSET_D

$THETA 0.125458 ; 11. SMOKCOV

$THETA -0.63924 ; 12. REGION 1

$THETA -0.605932 ; 13. REGION 3

$THETA -0.19589 ; 14. REGION 4

$THETA -0.408586 ; 15. REGION 5

$THETA -0.981896 ; 16. REGION 6

$OMEGA 0.449621 ; 1. iiv_tprog_T

$OMEGA BLOCK(3)

0.889033 ; 2. iiv_pmax_T

0.061607 0.365309 ; 3. iiv_offset_T

0 -0.0749947 1.09057 ; 4. iiv_baseline_T

$OMEGA 0.459249 ; 5. iiv_tprog_D

$OMEGA BLOCK(3)

0.984106 ; 6. iiv_pmax_D

-0.152554 1.47297 ; 7. iiv_baseline_D

0 -0.0797621 0.42101 ; 8. iiv_offset_D

$SIGMA 1 FIX ; 1. sigma1 PSI

$SIGMA 0.321843 ; 2. sigma2 PSI

$ETAS FILE=run228.phi ; ETAS from model without estimating covariate effect

$ESTIMATION MAXEVAL=9999 METHOD=1 INTER PRINT=1 NSIG=3 NOABORT

MCETA=1000 MSFO=msf229.msf

$COVARIANCE PRINT=E UNCONDITIONAL


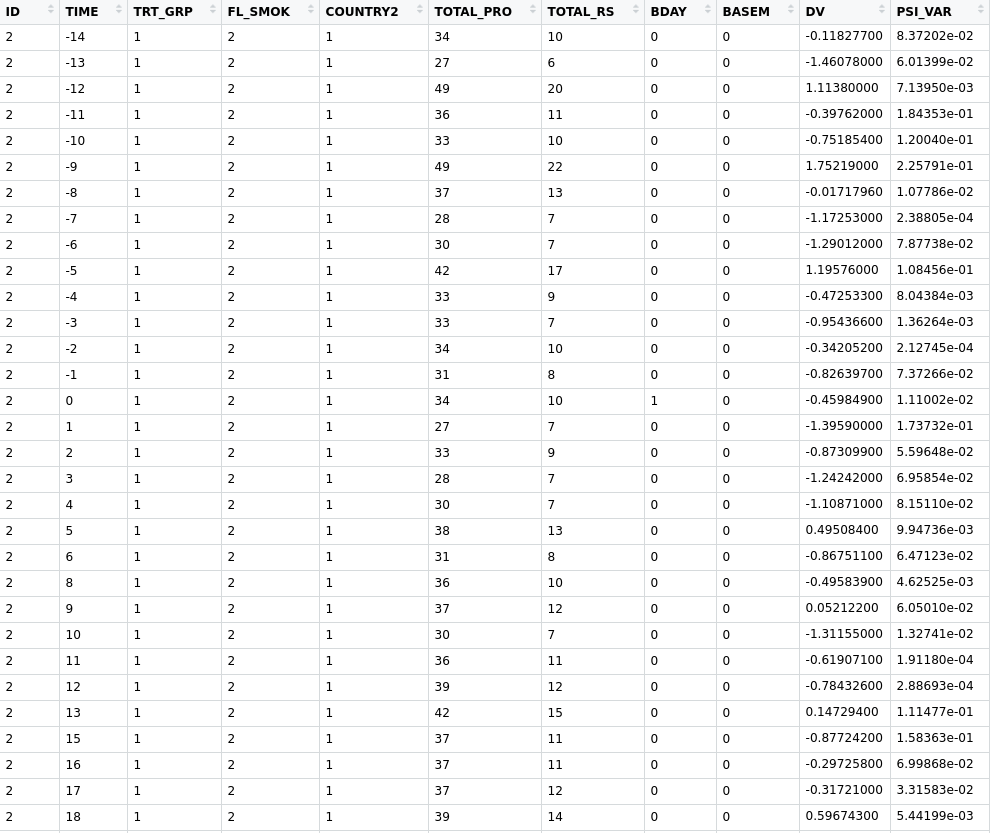

Supplement: Supplementary file 1 — (DOCX 5423 kb) [file 11095_2022_3194_MOESM1_ESM.docx]
